# Heterogeneity and Memory Effect in the Sluggish Dynamics of Vacancy Defects in Colloidal Disordered Crystals and Their Implications to High‐Entropy Alloys

**DOI:** 10.1002/advs.202205522

**Published:** 2022-10-30

**Authors:** Chor‐Hoi Chan, Qingxiao Huo, Anupam Kumar, Yunhong Shi, Huihui Hong, Yitong Du, Simiao Ren, Kin‐Ping Wong, Cho‐Tung Yip

**Affiliations:** ^1^ Faculty of Science Harbin Institute of Technology Shenzhen Shenzhen 518055 China; ^2^ Department of Applied Physics Hong Kong Polytechnic University Hung Hom Hong Kong China; ^3^ Present address: Department of Electrical and Computer Engineering Duke University Durham NC27705 USA

**Keywords:** colloidal crystal, glass, high‐entropy alloy, vacancy dynamics

## Abstract

Vacancy dynamics of high‐density 2D colloidal crystals with a polydispersity in particle size are studied experimentally. Heterogeneity in vacancy dynamics is observed. Inert vacancies that hardly hop to other lattice sites and active vacancies that hop frequently between different lattice sites are found within the same samples. The vacancies show high probabilities of first hopping from one lattice site to another neighboring lattice site, then staying at the new site for some time, and later hopping back to the original site in the next hop. This back‐returning hop probability increases monotonically with the increase in packing fraction, up to 83%. This memory effect makes the active vacancies diffuse sluggishly or even get trapped in local regions. Strain‐induced vacancy motion on a distorted lattice is also observed. New glassy properties in the disordered crystals are discovered, including the dynamical heterogeneity, the presence of cooperative rearranging regions, memory effect, etc. Similarities between the colloidal disordered crystals and the high‐entropy alloys (HEAs) are also discussed. Molecular dynamics simulations further support the experimental observations. These results help to understand the microscopic origin of the sluggish dynamics in materials with ordered structures but in random energy landscapes, such as high‐entropy alloys.

## Graphical abstract

Nonuniform distribution of particle size brings glassy properties to colloidal crystal systems. The vacancy dynamics of such disordered crystals are studied by experiments and molecular dynamics simulations. Heterogeneity and memory effect are observed in the sluggish vacancy dynamics. The results are further connected to the sluggish diffusion in high entropy alloys (HEAs).

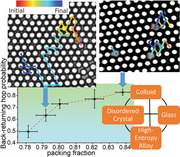

## 1 Introduction

Vacancy defect has long been known to be the major factor affecting the diffusion in metals and alloys, which in turn affects the thermal, mechanical, and transport properties of the materials. The difficulty of vacancy accumulation under stress for forming microvoids has recently been known to account for the strong fracture resistance of high‐entropy alloys (HEAs).^[^ ^1^ ^]^ Theorists are also aware that understanding the vacancy dynamics of HEAs can help us to understand its most controversial core effect,^[^ ^2^, ^3^, ^4^ ^]^ the sluggish diffusion effect.^[^ ^5^, ^6^, ^7^, ^8^, ^9^, ^10^ ^]^ As vacancy‐like defects have recently been shown to be important in glassy systems,^[^ ^11^, ^12^, ^13^, ^14^, ^15^ ^]^ the study of vacancy dynamics sounds useful in both crystalline and amorphous materials.

Currently, experimentally imaging vacancy (or atom) migration in a 3D structure is still unavailable. Observing vacancy (or atom) trajectories is achievable in 2D systems,^[^ ^16^, ^17^, ^18^ ^]^ but it is usually done by placing the target 2D system as adatoms on the surface of another substrate material, where the substrate can cause anisotropy in the vacancy trajectories.^[^ ^18^ ^]^ Some researchers thus prefer using colloids to model vacancy and interstitial migrations in crystals.^[^ ^19^, ^20^, ^21^, ^22^ ^]^ The “slow” movements of colloidal particles enable easy tracking of individual particle movements, making colloids useful for modeling the crystallization and melting process of materials.^[^ ^23^, ^24^, ^25^, ^26^, ^27^ ^]^ On the other hand, the structural relaxation in colloidal glass experiments is several orders “faster” than that in typical molecular dynamics simulations of glassy systems,^[^ ^28^ ^]^ turning colloid into a supreme tool to model the particle dynamics of glass.^[^ ^11^, ^29^, ^30^, ^31^, ^32^ ^]^

A former experiment has reported glassy‐like vibrational spectrums in colloidal crystal systems.^[^ ^33^ ^]^ Compressible colloids are used in that work, bringing a 2% difference in the mean separation between neighboring colloids. This causes nonuniform interactions between particles and results in glassy‐like vibrational spectrums. Simulations further reveal that increasing particle‐size polydispersity turns a system from crystal to disordered crystal and then to glass.^[^ ^34^ ^]^ The disordered crystal maintains a crystalline structure but exhibits glassy behaviors in the vibrational spectrum and mechanical properties.^[^ ^34^ ^]^ While atomic crystals that exhibit glass‐like thermal conductivity are believed to have useful applications in industry,^[^ ^35^ ^]^ such a class of system may have further practical applications and may serve as a simpler tool to understand the more complicated structural disordered glassy system.

In this work, we have three themes: I) We experimentally study the density‐dependent sluggish vacancy dynamics in colloidal disordered crystals. II) We demonstrate that disordered crystals with vacancies exhibit glassy properties that have not been discussed in the literature. III) We discuss the similarities between HEAs and disordered crystals through experiments and molecular dynamics simulations, and suggest our results of returning hop induced kinetic arrest in vacancy dynamics help to understand the microscopic origin of the controversial sluggish diffusion effect in HEAs.^[^ ^5^, ^6^, ^7^, ^8^, ^9^, ^10^ ^]^

## 2 Results and Discussion

### 2.1 Quasi‐2D Experiments

We perform optical video microscopy experiments on quasi‐2D colloidal systems,^[^ ^30^, ^36^ ^]^ using PMMA spheres with a mean diameter (σ) of 3.83 µm and a standard deviation (σ_SD_) of 0.17 µm.^[^ ^19^, ^20^, ^21^, ^22^, ^23^, ^24^, ^25^, ^26^, ^32^ ^]^ Our unimodal size distribution of colloidal particles enables the crystalline structure to emerge easily when the packing fraction (ϕ) is high. The increase of ϕ in the colloidal system corresponds to a decrease in the temperature of a modeled material.^[^ ^29^, ^30^ ^]^ Twenty three samples from medium to high density (ϕ ≈ 0.78–0.84) are continuously observed for 0.97 to 5.94 days, which is much longer than the typical observation time of less than a few hours in other colloidal experiments.^[^ ^19^, ^20^, ^21^, ^22^, ^23^, ^24^, ^25^, ^26^, ^29^, ^30^, ^31^, ^32^ ^]^ As each sphere has a slightly different size, the colloidal crystal system is similar to materials composed of many different elements, with each ion center having a different atomic size sitting in a random energy landscape, such as HEAs.^[^ ^4^ ^]^ We focus on the dynamics of the vacancies inside the colloidal crystals, which are surrounded by six nearest neighboring particles.

### 2.2 Sixfolded Symmetric Vacancies

**Figure 1:**
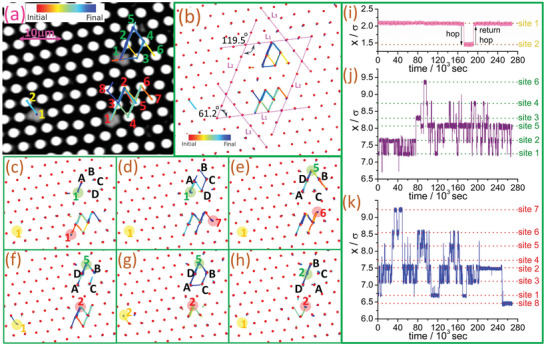
Sluggish vacancy dynamics in a high‐density colloidal disordered crystal. a) Experimental image at *t* = 0 superimposed with the vacancy trajectories for a ϕ = 0.837 sample under 3.11 days of observation. The displayed particle size is tunable under the microscope. Three vacancies are recorded as three grey area patches. The colored numbers labeled next to the lattice sites denote the visiting order of the three vacancies. The vacancy sitting close to the yellow numbers on the left side is an inert vacancy, whereas the vacancies sitting close to the green and red numbers on the right side are two active vacancies. b) Particle full‐time trajectories. Parallel magenta dotted lines of equal length are drawn along the three crystal axes to estimate the distortion of the crystal structure. c–h) Particle trajectories (red‐initial; blue‐final) when the full observation time is split up into six consecutive equal intervals. Red dots denote the particles, colored patches denote the three vacancies, and A–D mark four selected particles at the start of each time interval. i–k) Normalized *x*‐positions of the vacancy in the lower left, upper right, and lower right of (a) versus time. These demonstrate the high‐ϕ (or low temperature) disordered crystal systems not only have inert vacancies that hardly hop to other lattice sites, but they also have regionally trapped active vacancies. Glassy properties like dynamical heterogeneity and cooperative rearranging regions are also found in the disordered crystal system.

**Figure** **1**a (Movie S1, Supporting Information) shows a truncated photo of a high‐density sample (ϕ = 0.837). A clear crystalline structure with distortions is formed. Three vacancies, shown as grey area patches and marked with colored number 1, are found in the region. They are roughly in sixfolded symmetry, clearly sitting on lattice sites for most of the time, except during the short instanton time when hopping motion takes place. They are different from the vacancies observed in the early works of Pertsinidis and Ling,^[^ ^19^, ^20^ ^]^ which are in split SV, or other twofolded or threefolded symmetric forms. Moreover, our lattice sites serve as metastable regions for both the vacancies and particles, whereas several particles surrounding each vacancy in Pertsinidis and Ling are pushed away from the lattice sites.^[^ ^19^, ^20^ ^]^ Zhang and Liu have successfully created mono‐vacancies in sixfolded symmetry,^[^ ^21^ ^]^ but their mono‐vacancies are unable to hop to other lattice sites within the observation time.

### 2.3 Inert Vacancies

The three vacancies in Figure 1a all show sluggish dynamics in the 3.11 days of observation, but they behave in two different ways. The vacancy in the lower left part, marked with yellow numbers, represents one kind of sluggish dynamics. This vacancy has only hopped two times, back and forth, between two sites throughout the observation time (see Figure 1i). In the same sample, we find two other isolated vacancies sitting on the original lattice sites all the time. This type of vacancy is inert and seldom hops.

### 2.4 Active but Regionally Trapped Vacancies

The two vacancies in Figure 1a, marked with green and red numbers, represent another kind of sluggish dynamics. These vacancies are very active. The upper vacancy has hopped 190 times, while the lower right vacancy has hopped 230 times within the same observation time, counting only the cases of vacancy that have completely moved from one site to another. However, they are trapped in small regions, with the upper vacancy only hops between six lattice sites (green numbers), and the lower right vacancy only hops between eight lattice sites (red numbers). From Figure 1c–h, these two vacancies have reached their furthest sites quite early, but afterward, they just move between different sites they have visited before. Only at a very late time, the lower right vacancy moves to a new lattice site, red 8, which is only two sites away from the initial site. Figure 1j,k show the vacancies spend unequal time on each lattice site, and it is common for the vacancy to first hop to a new site, then stay there for a certain time and hop back to the previous site in the next hop. We call it a back‐returning hop. The back‐returning hop probabilities (*P*_ret_) are 73.2% and 73.9% for these two vacancies, which are significantly high values. Notice also that these two active vacancies are sometimes separated by only one particle, but they have never combined (see Figure S2, Supporting Information).

### 2.5 Anisotropic Motions and Simple Strain Test

The back and forth hops occur most frequently between sites green 1–2 for the upper vacancy and between sites red 2–3 for the lower right vacancy. These movements are both along the same crystal axis on the triangular lattice. A simple strain test is thus performed in Figure 1b, but it shows no significant elongations in any of the three crystal axes, which implies that the anisotropic motions should not be caused by the strain and stress in this region. Nevertheless, the crystal axes cross each other at angles 61.2° and 119.5°, which still signals a distortion in the triangular lattice structure due to particle size dispersion.

### 2.6 Localized Mobile Regions

Figure 1c–h also show that four particles near the vacancy initially arranged as A–B–C–D in the clockwise direction, are finally arranged as A–D–B–C after a long time. Meanwhile, they also move to different lattice sites. This demonstrates that the local movement of a vacancy brings a regional change in particle positions over a long time, while other particles sitting far away from the vacancies can only stay in the same lattice sites. This is similar to the dynamical heterogeneity of particle dynamics in a glassy system,^[^ ^30^, ^31^ ^]^ which has mobile particles moving around the cooperative rearranging regions (CRR),^[^ ^32^ ^]^ and at the same time has many immobile particles resting in the remaining part of the system. (Figure S14, Supporting Information illustrates a CRR in a colloidal glass.) A recent colloidal glass experiment and hard‐disk simulation have demonstrated that the motions in the CRR of high‐ϕ samples can be decomposed into string‐like motions happening at different time, accomplished with the transport of quasi‐voids, which move in the opposite directions relative to the hopping particles.^[^ ^11^ ^]^ While most of the time, the hopping motions in Figure 1 involve only one particle hop, occasionally, the vacancy takes a big hop to a site two steps away, accomplished with two particles moving together in the opposite direction. This is similar to the micro‐strings observed in glassy systems.^[^ ^37^ ^]^

### 2.7 Memory Effect in Diffusive Vacancies

**Figure 2:**
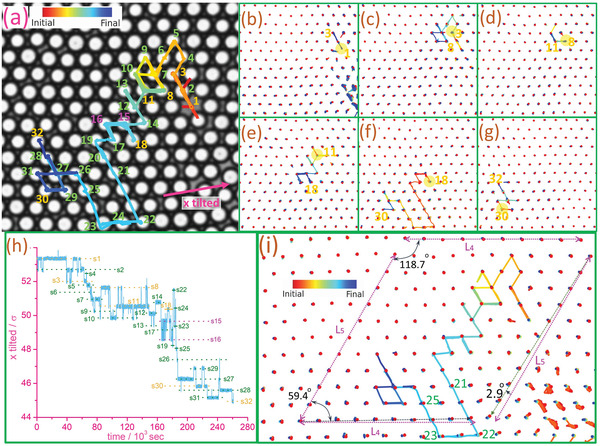
Sluggish vacancy diffusion in a medium‐density colloidal disordered crystal. a) Experimental image at *t* = 0 superimposed with the vacancy trajectory for a ϕ = 0.792 sample under 3 days of observation. b–g) Particle trajectories are split up over six consecutive equal time intervals, with red denoting initial motions and blue denoting final motions in each box. In each time interval, a circular yellow patch denotes the initial vacancy location, while orange numbers denote the start and final positions of the vacancy. h) Normalized positions of the vacancy along the *x*‐titled axis versus time. sN are the site numbers labeled in (a). The vacancy diffuses across many sites, spending unequal time in different sites, and hops back and forth frequently between several pairs of sites like 1–2, 8–11, and 15–16. This is a memory effect, which is also a property of glass. The vacancy spends only a short time between sites 19 and 27, and big hops consist of several particles moved together coherently are made between sites 21–22 and 23–25. i) Particle full‐time trajectories. Two parallel pairs of magenta dotted lines with equal length are drawn. Nine red dots (initial particle locations) in the upper region just fit L_4_, while nine red dots in the lower region spare a distance shorter than L_4_ by 3%. A 2.9° bending of particle alignment is found around the line L_5_ drawn at the right‐hand side. This shows a strain‐induced vacancy motion on a distorted lattice.

**Figure** **2**a (Movie S2, Supporting Information) shows a truncated photo of a sample with medium density (ϕ = 0.792). A vacancy, initially at site 1, has moved across more than 30 lattice sites in the 3 days of observation. The trajectories of this vacancy in Figure 2a–g look like an unbiased random walk, which is usually assumed in the perfect crystal structure, and has been observed in the mono‐vacancy migration in a sandwiched monolayer of MoS_2_,^[^ ^16^ ^]^ as well as in the di‐vacancy migration in a 2D graphene experiment.^[^ ^17^ ^]^ However, the vacancy in Figure 2 indeed hops between lattice sites with $P_{\mathrm{ret}}=54.4\%$, which is significantly larger than 1/6 as predicted for the random walk process on a triangular lattice. This is a memory effect. A former colloidal crystal experiment has also reported mono‐vacancies hopped with $P_{\mathrm{ret}}\approx 40\%$,^[^ ^19^ ^]^ but its vacancies are in twofolded or threefolded symmetry, which has preferred directions for vacancy hops. Our vacancy is on the other hand in sixfolded symmetry. Figure 2h further shows the detailed movement of the vacancy against time. Particular strong back and forth hopping motions are found between several pairs of lattice sites, which are not all along one crystal axis. This memory effect makes vacancy diffuses sluggishly. We believe it is caused by the nonuniformity of particle size, which resembles a random energy landscape in high‐entropy materials. Notice that the memory effect mentioned here differs from that defined in Pertsinidis and Ling,^[^ ^19^ ^]^ which is related to certain particles surrounding the vacancies not sitting on the lattice sites in their damped system. (Damping effect is further discussed in Supporting Information.) It should also be pointed out that the memory effect is a typical property of glass.^[^ ^29^, ^31^, ^38^ ^]^

### 2.8 Strain‐Induced Vacancy Motion on a Distorted Lattice

By comparing the lattice constant along the horizontal crystal axes on the upper side and the lower side of Figure 2i, a 3% difference is found. That means there is either an elongation across the particles on the upper side or a compression across the particles on the lower side. The crystal axis in another direction also shows a clear 2.9° of bending. These imply that stress and strain exist in this region, which should account for the overall drift of the vacancy from the upper right side to the lower left side.

### 2.9 Probabilities of Hopping Back Versus Packing Fractions

**Figure 3:**
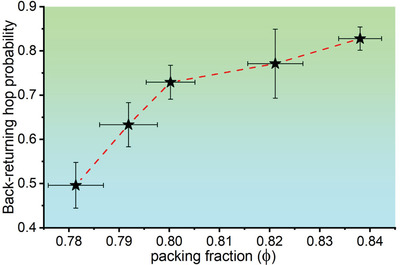
Back‐returning hop probability of vacancy (*P*_ret_) versus mean packing fraction of grouped samples. Only vacancy has first hopped from one lattice site to another lattice site, and then hops back from the new site to the previous site in the next hop, is considered as a back‐returning hop. 23 samples are grouped into 5 groups as stated in Section 4, such that each group has 6–21 vacancies and the total vacancy hops counted in each group is 379 to 2081. Similar increasing trends in *P*_*ret*_ are also found in other glassy systems.^[^ ^11^, ^39^, ^40^ ^]^

The heterogeneities in the dynamics of our vacancies found within the same sample and between different samples are further given in Figures S12 and S13, Supporting Information. **Figure** **3** shows the vacancy statistics when the 23 samples are grouped into 5 groups. An increasing trend of *P*_ret_ against ϕ is found, from 50% to 83%. This is similar to the recent experimental and simulation results performed in colloidal glass,^[^ ^11^ ^]^ as well as the simulation results in polymer glass and the lattice glass model,^[^ ^39^, ^40^ ^]^ if we recall the correspondence between ϕ and the inverse of the temperature of a modeled material.^[^ ^29^, ^30^ ^]^

### 2.10 Implications to Systems with Crystalline Structures but in Random Energy Landscapes

A lattice model with a random energy landscape and the presence of vacancies has shown similar glassy properties we have observed in our system.^[^ ^15^, ^40^ ^]^ Moreover, in molecular dynamics simulations of glass, either using different types of atoms with different interaction strengths,^[^ ^28^ ^]^ or using hard disks (or spheres) with different sizes,^[^ ^11^, ^30^, ^41^ ^]^ gives similar results. Therefore, we can assume the variation of particle radii resembles a random energy landscape, and we believe our results are applicable to materials with crystalline structures but in random energy landscapes.

#### 2.10.1 High‐Entropy Alloy

**Figure 4:**
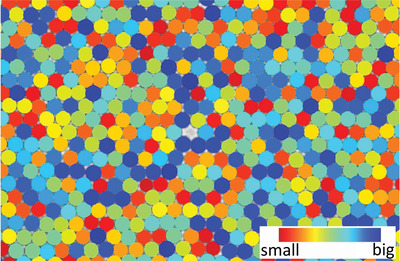
A selected region of our second highest density sample (ϕ = 0.838). The colloidal particles are colored from red to blue according to their estimated size (The method is given in Supporting Information) The suppression of arranging as intermetallic phase such as a big‐small‐big‐small… form illustrates our colloidal disordered crystals possess a property similar to the high‐entropy effect found in high‐entropy alloys.^[^ ^5^, ^42^ ^]^

One example is the high‐entropy alloy (HEA), which is defined as an alloy composed of five or more major elements in near‐equimolar ratios,^[^ ^4^, ^43^ ^]^ or with concentrations between 5% and 35%.^[^ ^6^ ^]^ HEAs exhibit four core effects: high‐entropy effect, severe lattice distortion, sluggish diffusion, and cocktail effects.^[^ ^4^, ^5^, ^6^ ^]^ One significant consequence of the high‐entropy effect is the suppression of the intermetallic phase, and the emergence of the solid solution phase.^[^ ^5^, ^42^ ^]^ If particles in our samples are arranged as big‐small‐big‐small…, the system will resemble a compound in an A–B–A–B–… form in the intermetallic phase.^[^ ^42^ ^]^ However, our 23 samples do not show such particle size‐dependent structure arrangement as shown in **Figure** **4**. This is similar to a HEA in a single solid solution phase.^[^ ^42^ ^]^ Severe lattice distortion means the size difference in the ionic centers causes distortions in the lattice. The crystalline structures of our samples, as shown in Figures 1 and 2 (and Supporting Informatio), also show distortions, and the magnitude is comparable to the recent experimental images of HEAs obtained from TEM and STEM.^[^ ^44^, ^45^, ^46^, ^47^, ^48^ ^]^ Sluggish diffusion means the composite atoms of the HEAs diffuse in the alloy at slower rates compared to the diffusion in their pure element forms.^[^ ^5^, ^6^, ^7^ ^]^ Both the existence and the underlying principles of this core effect are still under hot debate.^[^ ^2^, ^3^, ^4^, ^5^, ^6^, ^7^, ^8^, ^9^, ^10^ ^]^ Our results of vacancies diffuse with a strong memory effect, spending unequal time in different sites, and hopping back and forth frequently between pairs of sites should provide an excellent picture to understand the sluggish diffusion. Our finding that vacancies actively hop between different lattice sites, but get trapped in small local regions, making some particles only move in small local regions, should be a new way to understand the sluggish diffusion and a new phenomenon worth researchers in HEAs looking for.

#### 2.10.2 Simulations

**Figure 5:**
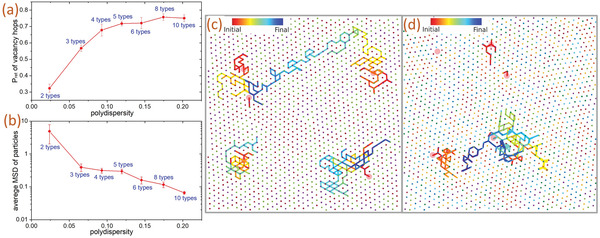
Simulation results for 90 2D disordered crystal systems composed of 2, 3, 4, 5, 6, 8, or 10 types of particles with increasing polydispersities but having the same packing fraction. a) Back‐returning hop probability of vacancy (*P*_ret_) versus polydispersity of the disordered crystal. The values of *P*_ret_ for all cases are greater than the theoretical value of 1/6 for a crystal composed of one type of particle. A general increasing trend is observed in this range of polydispersity. b) The average value of the mean square displacement (MSD) of particles versus polydispersity of the simulated system at 1.1 × 10^6^ Lennard–Jones time units. The MSD of particles decreases when the polydispersity increases, showing the sluggish diffusion effect is more prominent when the disordered crystal is more glassy. c,d) Trajectories (red‐initial; blue‐final) of five mono‐vacancies (red circular patches) created by removing five particles respectively from equilibrated systems composed of 1600 particles with c) three types of particles having a polydispersity of 0.066 and d) eight types of particles having a polydispersity of 0.175. The small dots denote the initial positions of particles and different colors represent different particle types. The mono‐vacancies created in a system composed of three types of particles generally diffuse further away compared to those in a system composed of eight types of particles. The lattice structure in the latter case is showing larger distortion as well. Some vacancies have been absorbed by the background at an earlier time compared to the other vacancies, such that their trajectory lines are not ended in blue. Both (c) and (d) show that these systems do not exist as an intermetallic phase.^[^ ^5^, ^42^ ^]^ These results show that the disordered crystals exhibit glassy properties and three core effects found in high‐entropy alloys. (Details of the simulations and the statistics are given in Supporting Information).

As a further discussion between HEAs and disordered crystals, we simulated 2D disordered crystals composed of 2, 3, 4, 5, 6, 8, or 10 types of particles with increasing particle‐size polydispersities but having the same packing fraction under the same temperature using a short‐range interaction potential. Five particles are removed from each of these equilibrated systems, initially composed of 1600 particles, to study the vacancy dynamics. **Figure** **5**a shows the back‐returning hop probability of vacancy (*P*_ret_) generally increases with the increase of polydispersity. The values of *P*_ret_ for all cases are significantly greater than the theoretical value of 1/6 for a pure crystal composed of only one type of particle which has a polydispersity of 0. Here, systems composed of more than four types of particles can be regarded as HEA systems,^[^ ^4^, ^43^ ^]^ and a disordered crystal system is getting closer to a glassy system as polydispersity increases.^[^ ^34^ ^]^ Figure 5b shows the mean square displacement (MSD) of particles decreases with the increase of polydispersity of a system. Here, the MSD is defined as the average of the square of the particle displacements between the end and the beginning of the simulation. A smaller value of MSD observed at a certain time means the diffusion rate of a system is smaller. The MSDs of systems composed of five to ten types of particles are over ten times smaller than those systems composed of only two types of particles. This demonstrates sluggish diffusion and the effect is more prominent when the disordered crystal is more glassy. Figure 5c,d show the trajectories of five mono‐vacancies created on disordered crystals composed of three and eight types of particles respectively. Vacancies created in the more polydisperse systems generally visit smaller numbers of lattice sites and hop with higher back‐returning probabilities. They are also easier to be absorbed by the background immediately after they are created. Lattice structures in both Figure 5c,d show severe lattice distortions and the effect is more prominent in the more polydisperse system. Denoting different particle types with different colors in Figure 5c,d, particles in both systems are not arranged as any forms of compounds but exist in the solid solution phase. The results in Figure 5 thus show that the disordered crystals demonstrate three core effects of high‐entropy alloys: high entropy effect, severe lattice distortion effect, and sluggish diffusion effect.^[^ ^4^, ^5^, ^6^, ^42^ ^]^

## 3 Conclusion

In conclusion, we have experimentally studied the vacancy dynamics in colloidal disordered crystals with different packing fractions. Vacancies exhibit heterogeneity in the dynamics, where some hardly hop to other lattice sites, and some actively hop between different sites but are localized in small regions or diffuse with a strong memory effect. These make them sluggish and have a very low diffusion rate. While the usual explanation for a slower diffusion rate found in a higher packing fraction (or lower temperature) system is that the vacancies and particles getting more difficult to hop from one lattice site to another, these findings provide additional reasons for the slow diffusion rate. We point out several similarities between glass and colloidal disordered crystal with vacancies present, including the dynamical heterogeneity, the presence of cooperative rearranging regions, memory effect, and the monotonically increasing back‐returning hop probability (up to 0.83) against the increase of packing fraction. In addition, we observe vacancy dynamics under strain. We believe the trajectory pictures and the sluggish dynamics found in our experiments are applicable to real materials that are structurally ordered but in random energy landscapes. We have pointed out four similarities between high‐entropy alloys (HEAs) and our colloidal disordered crystals and have verified that by simulating crystals composed of several types of particles with different diameters. The molecular dynamics simulations also show that the back‐returning hop probability and the sluggish diffusion effect increase with the increase of the particle‐size polydispersity, that is, more glassy. We further suggest that our results help in understanding the controversial sluggish diffusion in HEAs. Finally, we hope this work can raise attention to the interconnection between colloidal science, high‐entropy alloys, glassy physics, and defect physics.

## 4 Experimental Section

### Materials and Experiment

Poly(methyl methacrylate) spheres (manufacturer: microparticles GmbH, product name: PMMA‐R‐3.8, batch number: PMMA‐R‐B1167, solids content: 10% of aqueous suspensions) were immersed in water and sandwiched between two well‐cleaned glass plates, such that quasi‐2D systems were formed. The big particle size made the colloidal spheres heavy enough to sink to the bottom of the water solvent, and easily arrange as a monolayer to form quasi‐2D systems. Bigger particles also moved slower which enabled better particle tracking. Optical video microscopy measurements were made using two different microscopes, Leica DM2700M and OPS A200B, with oil immersion objectives and CCD cameras mounted to their eyepieces.^[^ ^30^, ^36^ ^]^ Digital photos were taken as 2 to 5 s per frame and recorded in 2560 × 1920 pixels or 1980 × 1114 pixels.

### Packing Fraction (ϕ) Estimation

The areas (*A*) recorded by the two microscopes were measured to be 46838.42 and 38413.83 µm^2^ with the help of a microruler. Following a common method in the literature,^[^ ^32^ ^]^ the packing fraction (ϕ) of each sample was estimated from

(1) $$\phi =N\pi \sigma^{2}/\left(4A\right)+N\pi \sigma_{\mathrm{SD}}^{2}/\left(2A\right)$$

where *N* is the number of particles recorded in the photo. The nontrivial second term added in Equation (1) was a small correction due to the nonuniformity of particle diameters. Each particle sitting at the edge of the photo was counted as half a particle in *N*. However, for one sample with obviously more boundary particles having half of their bodies inside the photo, each of its boundary particles were counted as 3/5 in *N*. Based on the observation, (3/5 − 1/2) × (Number of particles found at the edge of photo) was taken as the counting error of *N*. Twenty three samples from high to medium density, with packing fractions ϕ = 0.840 ± 0.003 to 0.778 ± 0.002 were used in this manuscript (refer to the central column of **Table** **1**). Each sample had around 2594 to 3259 particles recorded in each photo.

**Table 1:** Details of the statistics used in Figure 3

Mean ϕ of the group	ϕ of the composite samples	Vacancies selected	Total hops
0.838 (+0.005/−0.004)	0.840 ± 0.003, 0.838 ± 0.003, 0.837 ± 0.003, 0.837 ± 0.002	10	2081
0.821 (+0.006/−0.005)	0.824 ± 0.003, 0.821 ± 0.003, 0.818 ± 0.002	6	379
0.800 (+0.004/−0.005)	0.802 ± 0.002, 0.802 ± 0.002, 0.801 ± 0.002, 0.800 ± 0.002,	14	845
	0.798 ± 0.003, 0.797 ± 0.002		
0.792 (+0.005/−0.006)	0.796 ± 0.002, 0.792 ± 0.002, 0.788 ± 0.002	6	761
0.781 (+0.006/−0.005)	0.785 ± 0.002, 0.783 ± 0.002, 0.782 ± 0.002, 0.781 ± 0.002	21	1158
	0.781 ± 0.002, 0.779 ± 0.002, 0.778 ± 0.002		

### Equilibration Time and Observation Time

Each sample was left stationary under the microscope for around 1 to 2 days for equilibration. To ensure that the system has reached a quasi‐equilibrium state, the photos and plot the particle trajectories were processed to check if all the strong collective movements in the system had died out. After that, *t* = 0 was set and each sample was continuously observed for 0.97 to 5.94 days. The highest density sample with ϕ = 0.840 was observed in three different periods, making that sample had been observed for more than 13 days.

### Defining Vacancy Locations

For most of the observation time, vacancies were clearly sitting on different lattice sites. The precise position of a vacancy was defined by the average position of the six particles surrounding it. When a particle attempted to hop from one site to another, the average position of the eight neighbors surrounding the moving particle was used to define the location of the vacancy. In a few special cases, a vacancy made a big hop with more than one particle moving together in a string form, such that the vacancy directly moved to a location at two or three sites away. This was often found in the samples when a vacancy was hopping from the crystalline region into the amorphous boundary, as shown in Figures S8–S10, Supporting Information. In these cases, more particles were used to do the averaging. A program code was developed to track the trajectory of the vacancy as described in Figure S15, Supporting Information.

### Defining Hopping Motions

A movement was classified as a hop only when the vacancy had migrated successfully from one lattice site to another site and had stayed there for at least three frames. For a vacancy first hop was from site A to site B, and then it hoped back from site B to site A in the next hop, the second hop was classified as a back‐returning hop. If a vacancy attempted to hop to another site and had moved for some distance, but failed to reach the other lattice site, its motion will not be classified as a hop, nor will this attempt be included in the statistics of the back‐returning hop probability (*P*_ret_). Vibrational motions were not included in the statistics.

### Statistical Analysis

In this subsection, the details of the calculations of the back‐returning hop probabilities of vacancies (*P*_ret_) in Figure 3 are given. Each of the samples had one to ten vacancies surrounded by six nearest neighboring particles inside the crystal domains. Some vacancies were formed in the middle of the observation and were absorbed before the end of the observation. Sometimes, a vacancy was formed within a few lattice sites of a formerly absorbed vacancy. In this case, the two vacancies were classified as one vacancy that existed in different time periods. As only a few vacancies were found in the crystalline regions of each sample, the 23 samples were grouped into five groups, denoted by the mean values of their packing fractions (ϕ), such that each group had more vacancies and hops counted in the statistics. The details are shown in Table 1. Only mono‐vacancies that had hopped more than or equal to five times were included in the statistics. More vacancies and hops counted in a group means the *P*_ret_ calculated in Figure 3 is more reliable. The x‐error bars in that figure are the maximum difference between the mean ϕ of the groups and the ϕ ± errors of their composite samples. Each y‐value in Figure 3 is the direct average of the back‐returning hop probabilities (*P*_ret_) of the mono‐vacancies in the same group. The corresponding y‐error bar was the standard error, which was the standard deviation of all the *P*_ret_ in the group divided by the square root of the number of vacancies in the group. Further information about the vacancy statistics is given in Figures S12 and S13, Supporting Information.

## Conflict of Interest

The authors declare no conflict of interest.

## Author Contributions

C.H.C and Q.H. contributed equally to this work. C.T.Y. and C.H.C. suggested the major directions. Experiments were done by Q.H. and Y.S. with the help of H.H. and Y.D. Simulations were done by A.K. with the help of C.H.C. Analysis was done by C.H.C. with the help of Q.H., Y.S., H.H., and Y.D. Programming works were done by C.H.C., A.K., S.R., and K.P.W. The manuscript was prepared by C.H.C., mainly corrected by C.T.Y., and proofread by all authors. C.T.Y. supervised the whole work.

## Supporting information

Supporting Information

Supplemental Movie 1

Supplemental Movie 2

## Data Availability

The data that support the findings of this study are available from the corresponding author upon reasonable request.
