# Supplementary material for: Heterogeneity and Memory Effect in the Sluggish Dynamics of Vacancy Defects in Colloidal Disordered Crystals and Their Implications to High‐Entropy Alloys
Source: Adv Sci (Weinh). 2022 Oct 30;9(36):2205522. doi: 10.1002/advs.202205522 (PMC9799019; doi:10.1002/advs.202205522)
Supplement: Supplementary file 1 — Supporting Information [file ADVS-9-2205522-s001.pdf]

ADVANCED SCIENCE
Open Access

Supporting Information

for *Adv. Sci.*, DOI 10.1002/advs.202205522

Heterogeneity and Memory Effect in the Sluggish Dynamics of Vacancy Defects in Colloidal Disordered Crystals and Their Implications to High-Entropy Alloys

*Chor-Hoi Chan, Qingxiao Huo, Anupam Kumar, Yunhong Shi, Huihui Hong, Yitong Du, Simiao Ren, Kin-Ping Wong and Cho-Tung Yip**

Supplementary Information (SI) for 'Heterogeneity and Memory Effect in the Sluggish Dynamics of Vacancy Defects in Colloidal Disordered Crystals and their Implications to High-Entropy Alloys'

*Chor-Hoi Chan Qingxiao Huo Anupam Kumar Yunhong Shi Huihui Hong Yitong Du Simiao Ren Kin-Ping Wong Cho-Tung Yip**

Dr. C.-H. Chan, Q. Huo, Dr. A. Kumar, Y. Shi, H. Hong, Y. Du, Prof. C.-T. Yip
Faculty of Science,
Harbin Institute of Technology Shenzhen,
Shenzhen, China PC:518055
Email Address: h0260416@hit.edu.cn

S. Ren+, K.-P. Wong
Department of Applied Physics,
Hong Kong Polytechnic University,
Hung Hom, Hong Kong, China
+ Present address: Department of Electrical and Computer Engineering, Duke University, Durham, NC27705, USA

Keywords: *Colloidal Crystal, Glass, High-Entropy Alloy, Vacancy Dynamics*

This PDF file includes:

Section S1: Estimation of the particle size distribution
Section S2: Formation and absorption of vacancies in medium-density samples
Section S3: Method for comparing particle size order in Figure 4 of the main text
Section S4: Relation between viscosity effect and particle size
Section S5: Simulation details and more about the statistics in Figure 5 of the main text
Section S6: Tracking particle and vacancy locations
Section S7: Supplementary figures

Figure S1 explains how to estimate the particle size distribution.
Figure S2 shows different selected moments of the vacancies in Figure 1 of the main text.
Figures S3−S11 show more examples of the vacancies and defects, as well as the distorted crystalline structures obtained in the experiments.
Figure S12 shows the back-returning hop probabilities (P_{ret}) against the hopping frequencies of selected vacancies in six samples with different packing fractions. It further demonstrates the heterogeneity in vacancy dynamics.
Figure S13 shows the number of lattice sites visited by the vacancies in different samples.
Figure S14 illustrates the concept of dynamical heterogeneity in glass using an unpublished result from our previous colloidal glass experiment. [11]
Figure S15 illustrates the idea of our vacancy tracking code for the experimental data.

References 11, 19−21, 28, 29, 34, 42, 49−53

S1 Estimation of the Particle Size Distribution

We prepare all our experimental samples using the same colloids ordered from the same manufacturer. To estimate the diameter distribution of our colloids, a very low-density sample of packing fraction (ϕ) ≈ 0.56 with around 2300 particles is prepared, such that the space between particles can be seen clearly. Particles are recorded as white circles as light shined from the bottom of the samples, passing through the colloidal particles with little refraction. Due to total internal reflection at the boundary of the particle spheres, there is always a black edge surrounding each white circle. We adjust the focus of the microscope to

achieve minimal black edges. Image recognition software is then used to match those particle images as shown in Figures S1(a)−(c).[49] The pixel length of the photo against the actual distance is obtained by placing a micro-ruler under the microscope. The diameter of each particle is then measured in μm. The mean diameter is found to be $\sigma = 3.83$ μm and the standard deviation is $\sigma_{SD} = 0.17$ μm (i.e. 4.5% of σ). They are small enough to allow Brownian motions but are relatively big compared to those in typical colloid experiments. These values are further used for estimating ϕ of different samples. The manufacturer also measured the particle size distribution and determined a mean diameter of 3.77 μm and a standard deviation of 0.11 μm (i.e. 2.9% of their mean diameter). The difference between the measured values suggests that there may be a systematic error in the calculated packing fractions. However, the results are still justified as our interests lie in the change of dynamics against the change of packing fraction. Section S3 further introduces a method for comparing the order of particle size in the high-density samples.

S2 Formation and Absorption of Vacancies in Medium-density Samples.

In Figure 3 of the main text and Figures S12 & S13, the existence time of some vacancies is shorter than the observation time of their samples. In our samples with medium density ($\phi \lesssim 0.8$), as particles have more space to move, sometimes other defects or small disordered regions rearrange to form a crystalline structure with a vacancy surrounded by 6 particles. Former research has shown that the boundary regions between different colloidal crystal grains are amorphous.[50,51] Sometimes vacancies are formed at the amorphous boundary regions and diffuse into the crystals (see Figure S11 and *Yip et al.*).[11] Most of these vacancies later diffuse into the amorphous boundaries, change into other defects, or get absorbed when the crystalline structure rearranges to more disordered forms. (Figures S8−S11 show some trajectories.) On the other hand, at the grain boundaries of high-density samples, we neither see any formation nor absorption of 6-folded symmetric vacancies, but 5-folded symmetric point defects are easily seen over there (see Figures S3 & S5 for the 5-folded symmetric defects).

S3 Method for Comparing Particle Size Order in Figure 4 of the Main Text

In this section, we first introduce a new method for comparing the size order of particles in the high-ϕ experimental colloidal samples. This method is used in Figure 4 of the main text to prove that particles in our colloidal crystals are not arranged as an intermetallic phase,[42] such as a big-small-big-small-big-small... form. Then, we numerically build a high-density system with similar conditions to our sample in Figure 4 to test the accuracy of our method.

S3.1 Methodology

Figure S5 shows the same inert vacancy as that in Figure 4 of the main text. Using image recognition software,[49] we can obtain the position and the diameter of each white circle in the original photos. However, the true diameter of each particle is slightly bigger as a part of each particle is inside the black edge surrounding the white circle. Due to light distortion, the white circles observed in the central field of view of this microscope are generally slightly bigger than those found in the boundary region, i.e. the black edges surrounding the white circles are in general thicker in the boundary region. This is not distinguishable by human eyes when looking at Figure S5 but can be detected by computer software. Therefore, we do not directly compare the particle size order through the measured diameter of each white circle, $\sigma_{p,whitecir}$. Instead, as this high-density sample has particles packed quite closely together, we estimate the particle diameters by recursively increasing the diameters of all particles through

$$\sigma_{p,k}(t) = \sigma_{p,k-1}(t) + \Delta\sigma, \qquad \sigma_{p,0}(t) = \sigma_{p,whitecir}(t) \tag{S1}$$

where p is the particle label, k is the iteration step, t is the time frame of the photo considered, and $\Delta\sigma$ is a constant iteration step-size which is taken to be around 1% of the average $\sigma_{p,whitecir}$ detected. When a particle finds its estimated diameter, $\sigma_{p,k}(t)$, is going to touch or overlap any of its neighbors in the next

iteration step, its iteration stops but the iterations for the other particles continue. The iteration process is finished when every particle is going to touch another particle in the next iteration step. This process is carried out for the photo taken in every time frame, such that we get a slightly different estimated diameter for each particle in each frame. The time-averaged estimated diameter, $\sigma_{p,iter}$, is taken as the resultant estimated diameter of each particle, which is then sorted from small to big, and used for comparison in Figure 4 of the main text.

S3.2 Numerical Verification

S3.2.1 A Testing System

To test the accuracy of our method, we numerically build a triangular lattice with 40×40 lattice sites in periodic boundary conditions. Each lattice site is separated from its neighboring sites by the same distance, $r_{lattice}$. This distance is chosen to be the average distance between the centers of neighboring particles sitting in the crystalline regions of our experimental sample with $\phi = 0.838$, which is used in Figure 4 and Figure S5. We randomly assign 40×40 hard disks on these lattice sites, with their diameters following a normal distribution having their mean and standard deviation equal to the same σ and σ_{SD} of our colloids (given in Section S1). As the distance between lattice sites is just slightly bigger than σ, some allocated hard disks will overlap their neighbors, which is not allowed in a hard-disk system. Therefore, inspired by the recent SWAP Monte Carlo simulation, [28] we perform swaps between hard-disk pairs to change the system. The is done by:

(1) Randomly pick up two hard disks, p_1 and p_2.
(2) Count the total number of neighbors overlapped with p_1 or p_2, denote it as f_{old}.
(3) Exchange p_1 and p_2, and count the total number of neighbors overlapped with p_1 or p_2, denote it as f_{new}.
(4) If $f_{new} \leq f_{old}$, we accept the exchange. Otherwise, we reject the exchange and the two disks return to their previous positions.
(5) Repeat steps 1 to 4 for 2000000 times, and check the sum of f_{old} in every 10000 steps, denote it as f_{sum}. If the initial configuration is good, f_{sum} will decrease to 0 as time goes by. In the case f_{sum} does not decrease to zero, just regenerate the initial configuration and go through these steps again.

Afterward, hard disks of different sizes are found to be allocated randomly on the lattice sites without overlapping with each other. Then, we perform a normal Monte Carlo simulation for $100000 \times 40 \times 40$ steps, i.e. 100000 MCSS. In each step, a hard disk is randomly selected, and a direction is randomly chosen for it to move for a distance of $\Delta\sigma$ as defined after Equation S1. The move is accepted if the disk does not overlap with any of its neighbors in the new position. The system configuration is recorded after every 40×40 steps. In this way, we have generated a system with a known diameter for each composite particle, and the configurations are recorded for 100000 frames.

S3.2.2 Testing Particle Size Order

In the colloidal experiments, the diameters of the white circles recorded are smaller than their actual diameters. Therefore, we now assume that only an initial diameter, $\sigma_{p,initial}$, which is smaller than the true diameter, $\sigma_{p,true}$, is detected for each hard disk p prepared in the last subsection. Then, we apply our iteration method to the configurations recorded in each time frame, and obtain our resultant time-averaged estimated diameter, $\sigma_{p,iter}$, for each hard disk. As we are only interested in the size order of the hard disks, so we define a quantity to measure the relative size order difference between the estimated diameter, $\sigma_{p,iter}$, and the true diameter, $\sigma_{p,true}$, for each hard disk p:

$$\Theta_p = \frac{|size\ order\ of\ \sigma_{p,iter} - size\ order\ of\ \sigma_{p,true}|}{Total\ number\ of\ hard\ disks}. \tag{S2}$$

For example, if the smallest hard disk of our artificial system is estimated to be the 10th smallest hard disk, then $\Theta_p = |10 - 1|/1600$.

We use two different choices of $\sigma_{p,initial}$ to test the accuracy of our method. First, we consider all hard disks have the same constant $\sigma_{whiteavg}$, which is taken to be the average $\sigma_{p,whitecir}$ of all the particles detected in our colloidal experiment with $\phi = 0.838$. The mean and standard deviation of Θ_p are then 0.092 and 0.080. That means using this initial condition for the iteration, our estimated size order deviates from the true size order by around 9.2%. Moreover, we find only around 5% of hard disks have their estimated size order deviated from the true size order by more than 25%.

In our second case, we consider $\sigma_{p,initial} = \sigma_{p,true} - (\sigma - \sigma_{whiteavg})$, where σ is the mean diameter of our colloidal particles (or our hard disks). The mean and standard deviation of Θ_p are then 0.009 and 0.008. That means using this initial condition for the iteration, our estimated size order deviates from the true size order by around 0.9%. Moreover, we find only around 5% of hard disks have their estimated size order deviated from the true size order by more than 2.5%.

In the colloidal experiments, since bigger particles are generally recorded as bigger white circles, so our second test should be closer to the actual situation, and the result is very good. The first test can be taken as the error limit of our method, which is still a good result.

S4 Relation between Viscosity Effect and Particle Size

When a PMMA sphere is moving across an aqueous medium, small water molecules will exert a drag force to slow down the movement of the sphere. This force of viscosity is given by the Strokes' law as:

$$F \propto v\sigma_1 \tag{S3}$$

where σ_1 is the diameter of the PMMA sphere, and v is the flow velocity relative to the sphere. Here, we approximate v as the velocity of our moving sphere. As mass is proportional to σ_1^3, then by the Newton's 2nd Law, the acceleration contributed by the force of viscosity is:

$$a \propto v\sigma_1^{-2} \tag{S4}$$

The typical time for a colloidal sphere to diffuse a distance of its radius $\sigma_1/2$ is: [29]

$$t \propto \sigma_1^3 \tag{S5}$$

The average speed in this time interval can be taken as

$$v = \sigma_1/(2t) \propto \sigma_1^{-2} \tag{S6}$$

Inserting it back to Equation S4, we arrive:

$$a \propto \sigma_1^{-4} \tag{S7}$$

This means if a colloidal particle is bigger, its motion will be less affected by the drag force of the medium. The mean diameter of our PMMA sphere is 3.83 μm, which is over 10 times the mean diameter of the particles used in *Pertsinidis and Ling.* [19,20] Thus, the contribution from the drag force to change the velocities of our colloidal spheres is 10000 times smaller than that in *Pertsinidis and Ling.* [19,20] This shows that viscosity has a much smaller effect on our system compared to that in *Pertsinidis and Ling.* [19,20] Therefore, the strong damping effect observed in *Pertsinidis and Ling* is not found in our samples. [19]

S5 Simulation Details and More about the Statistics in Figure 5 of the Main Text

We consider two-dimensional crystal systems composed of $N = 1600$ particles interacting via short-range repulsive inverse power-law potential,

$$U_{ij} = \varepsilon(\frac{\sigma_{ij}}{r_{ij}})^{12} \tag{S8}$$

where r_{ij} is the separation between the two neighboring particles i and j, and $\sigma_{ij} = (\sigma_i + \sigma_j)/2$ is the average diameters of the two neighboring particles. The potential is truncated at a cutoff distance of $1.25\sigma_{ij}$ and ε is taken to be 1. We have studied systems composed of $2, 3, 4, 5, 6, 8$, and 10 types of particles, which have uniformly distributed particle diameters with a mean diameter of $\sigma \approx 1$. Defining polydispersity as the range of the uniformly distributed diameters divided by σ, [34] these systems have polydispersities of $0.024, 0.066, 0.093, 0.119, 0.145, 0.175$, and 0.202 respectively such that the polydispersity increases with the increase in the number of particle types. 10 to 16 independent systems are studied for each polydispersity and a total of 90 simulated systems are considered in Figure 5 of the main text.

We have performed molecular dynamics (MD) simulations using the open software LAMMPS. [52] The built-in Lennard-Jones units in LAMMPS are adopted such that temperature and time are measured in εk_B^{-1} and $m^{1/2}\sigma\varepsilon^{-1/2}$ respectively. Here, k_B is the Boltzmann constant and all the particles are set to have the same mass $m = 1$. A fixed time step size of 0.001 is adopted and the periodic boundary conditions are used throughout the MD simulations.

In the beginning, particles are randomly allocated on triangular lattices confined to 2-dimensional square boxes with adjustable lengths $L = 40$. The systems first evolve at a high temperature of $T = 10.0$ for 2×10^6 to 6×10^6 MD steps under NPT ensemble (constant number of particles, pressure, and temperature ensemble) in LAMMPS. Meanwhile, the values of pressure are adjusted multiple times for each system until all our systems have the same packing fraction of $\phi = 0.60$. For a system with J types of particles,

$$\phi = \frac{\pi}{4L^2}(N_1\sigma_1^2 + N_2\sigma_2^2 + ... + N_J\sigma_J^2) \tag{S9}$$

where $N_I \approx N/J$ is the number of particles of type I in the system and the simulation box length L has already been elongated. After this early melting process, the systems are already completely different from their initial configurations, but the crystal structure of these systems is still maintained. We then abruptly quench the systems to $T = 0.3$, and equilibrate our systems using hybrid MD simulations with Monte Carlo swaps under NVT ensemble (constant number of particles, volume, and temperature ensemble). [28] 2×10^7 to 4×10^7 MD steps are taken in the equilibration of each system and 10 MC swaps are attempted at every 200 MD steps. After the equilibration, we randomly remove 5 particles to create 5 vacancies on each system and further perform normal MD without swap in NVT ensemble at $T = 0.3$ to study the dynamics. 1.1×10^9 MD steps (i.e. $t_{total} = 1.1 \times 10^6$ in Lennard-Jones time unit) are taken and the particle positions in both instantaneous and time-coarsening forms are recorded every 4000 MD steps. The average position of each particle in 1000 successive MD steps is taken as the time-coarsening particle coordinate.

For the mono-vacancies created on the simulation systems, some exist throughout the simulation period while some exist for a time shorter than t_{total}. Some are even absorbed by the background immediately after they are created, like the vacancy in Figure 5(d) of the main text that has no trajectory line attached to it. Each data point in Figure 5(a) of the main text is the direct average of the back-returning hop probabilities (P_{ret}) of 36 to 48 mono-vacancies taken from 10 to 16 independent systems, whereas its error bar is the standard error. Only mono-vacancies that have hopped more than 5 times are included in the statistics. The number of hops made by each vacancy varies a lot. Among the vacancies that exist throughout the simulation period, some only hop for around 30 times while some hop more than 10000 times.

S6 Tracking Particle and Vacancy Locations

S6.1 Experimental Data

The center positions of the colloidal particles are extracted from the photos by an image processing code. [49,53] The photos are recorded frequently enough to capture a single hop event through multiple frames. A program code is developed to track the trajectory of the vacancy for the experimental data as described in Figure S15. Corrections are further made by comparing the results with the particle motions tracked in Ovito and the original photos. Subsequent statistics of the hopping motions can be obtained easily afterward.

S6.2 Simulation Data

Although the particle positions have been recorded in every 4 Lennard-Jones time units, the instanton time of these systems is comparable to or less than this time interval, such that if a particle (vacancy) hops at a certain time, the particle will move from one lattice site to another lattice site within one to three frames. A program code is developed to track the vacancy trajectories of the simulation data. It is based on comparing the position of each particle i at the current frame, $\mathbf{r}_i(t)$, with its positions at the three previous frames, i.e. $\mathbf{r}_i(t-1)$, $\mathbf{r}_i(t-2)$, and $\mathbf{r}_i(t-3)$. Whenever any of these displacements is greater than 0.6 and the recorded vacancy position is within a distance of 0.5 from $\mathbf{r}_i(t)$, the vacancy (and the particle) is defined to have hopped from one lattice site to another lattice site. Notice that the lattice constant of our systems is around 1.2. The position of a vacancy at frame t is defined as

$$\mathbf{R}(t) = \frac{\int_{t_a}^{t_b} \mathbf{R}_{temp}(t')dt'}{t_b - t_a}, \tag{S10}$$

where $t \in [t_a, t_b]$, with t_a is the time when the vacancy has hopped from the previous lattice site to the current lattice site and t_b is the time when the vacancy still stays in the current site before hopping to the next site. $\mathbf{R}_{temp}(t)$ is the average position of the six neighboring particles surrounding the vacancy at time t. If a vacancy only stays at a lattice site for less than 3 frames before the next hop (i.e. $t_b - t_a < 3$), we take

$$\mathbf{R}(t) = \frac{1}{2}[\frac{\int_{t_a}^{t_b} \mathbf{R}_{temp}(t')dt'}{t_b - t_a} + \mathbf{R}(t'')], \tag{S11}$$

where $\mathbf{R}(t'')$ with $t'' < t_a$ is the previous vacancy position obtained at the current lattice site.

Sometimes two to three neighboring particles hop concurrently to new lattice sites like the string-like motion in glassy systems, making the vacancy hop to a lattice site two to three sites away. To detect this kind of big hop, the $\mathbf{R}(t)$ obtained is further checked with other particles that hop at the current time t, and if another particle is found within a distance of 0.5 from $\mathbf{R}(t)$, then another six neighboring particles are used to re-define $\mathbf{R}_{temp}(t)$ and thus $\mathbf{R}(t)$. This process is taken recursively to shift the vacancy position until no particles are further found near the final value of $\mathbf{R}(t)$.

In the ideal situation, vacancies are all in a 6-folded symmetric form all the time. However, for those systems that have relatively big polydispersities, their vacancies are occasionally deformed, and a few of them have even temporarily changed to a 5-folded symmetric form. Moreover, sometimes a vacancy makes a big hop with two particles moved together, but one particle is temporarily not sitting on a lattice site. These sometimes bring errors to the subsequent tracking of the vacancy positions. Therefore, to ensure accurate tracking of the vacancy trajectory, the minimum distance between $\mathbf{R}_{temp}(t)$ and the six neighboring particles of the vacancy is checked in each frame. Whenever this distance is smaller than 0.7, particles in the first one or two neighboring shells of the recorded six neighbors of the vacancy are selected. If six of these selected particles have their 6th closest particles separated from them by distances more than 1.6, and if these six particles are arranged as a ring, then these six particles will be regarded as the six new neighboring particles of the vacancy and a corrected vacancy position will be obtained. The separations between vacancy positions at different lattice sites are further checked and minor corrections are further made by comparing the results with the particle motions tracked in Ovito to ensure accuracy. Both the instantaneous particle positions and the time-coarsening particle positions are used for analysis. They are considered separately as a cross-check of the results.

S7 Supplementary Figures

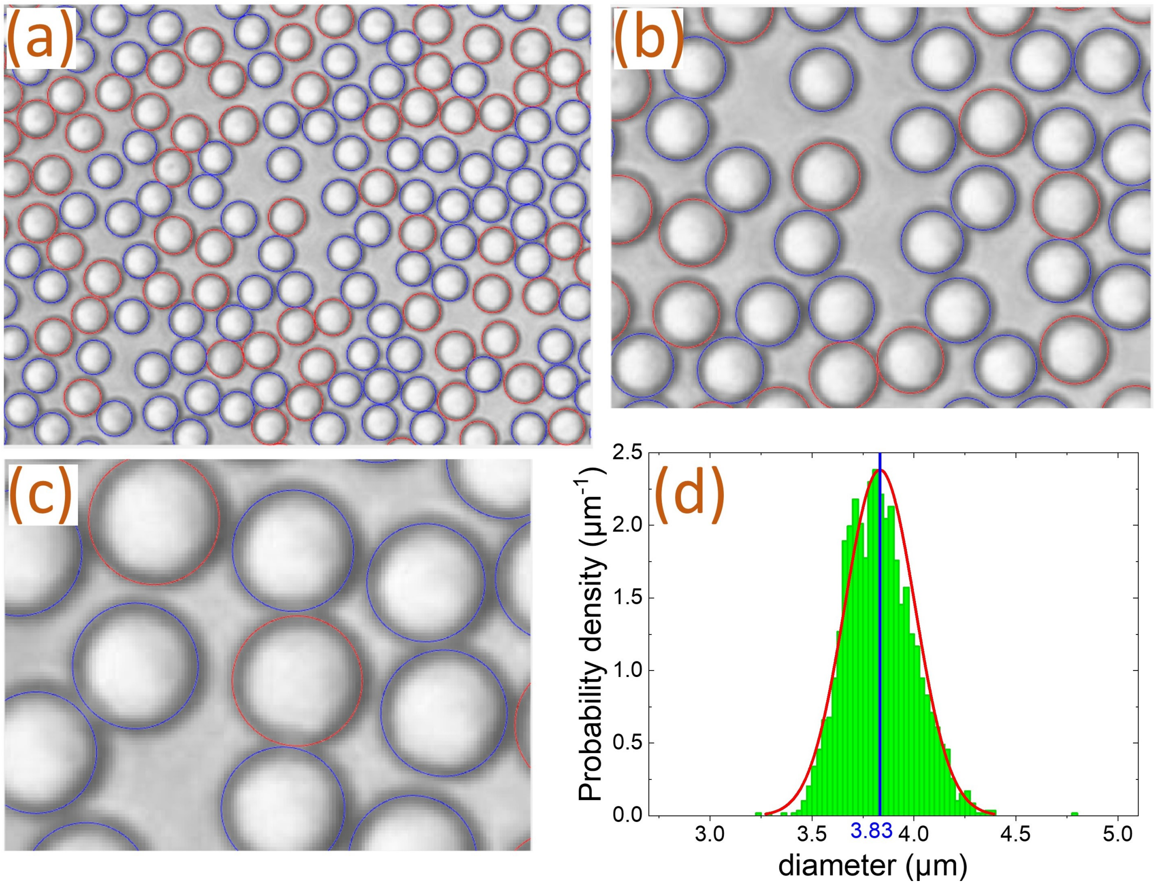

Figure S1: (a)−(c) Particle images of a very low-density sample with $\phi \approx 0.56$ in different magnifications. The photo is taken by the better microscope, Leica DM2700M. Image recognition software is used to match the particle images with red and blue circles. [49] We can see that the blue and red circles fit well to the middle of the black edges of the particle circles. The pixel length of the photo against the actual distance can be obtained by placing a micro-ruler under the microscope. The diameter of each particle in μm can then be calculated. (d) Overall particle size distribution. The mean diameter is found to be $\sigma = 3.83\ \mu m$, and our standard deviation is $\sigma_{SD} = 0.17\ \mu m$.

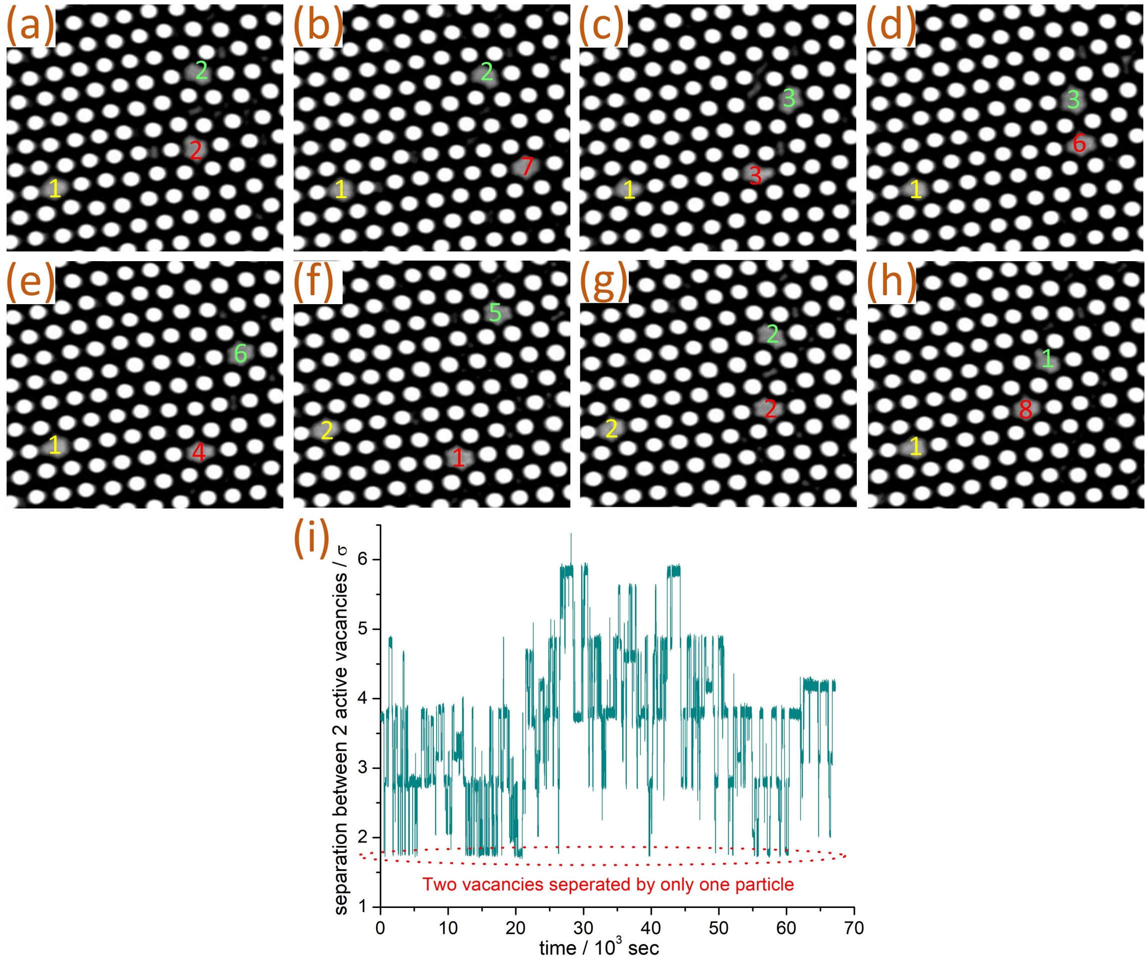

Figure S2: Selected moments of the three vacancies in Figure 1 of the main text, with $t =$ (a) 3200 sec, (b) 35200 sec, (c) 78400 sec, (d) 79200 sec, (e) 95200 sec, (f) 174800 sec, (g) 184000 sec, and (h) 266000 sec. The colored site numbers are the same as in Figure 1(a) of the main text. (i) shows the separation between the two active vacancies on the right-hand side against time. The two active vacancies are sometimes close together, sometimes far apart, but they have never clustered together nor have they pushed each other away. Moreover, their movements are localized in this regional area. Movie S1 shows their detailed movements.

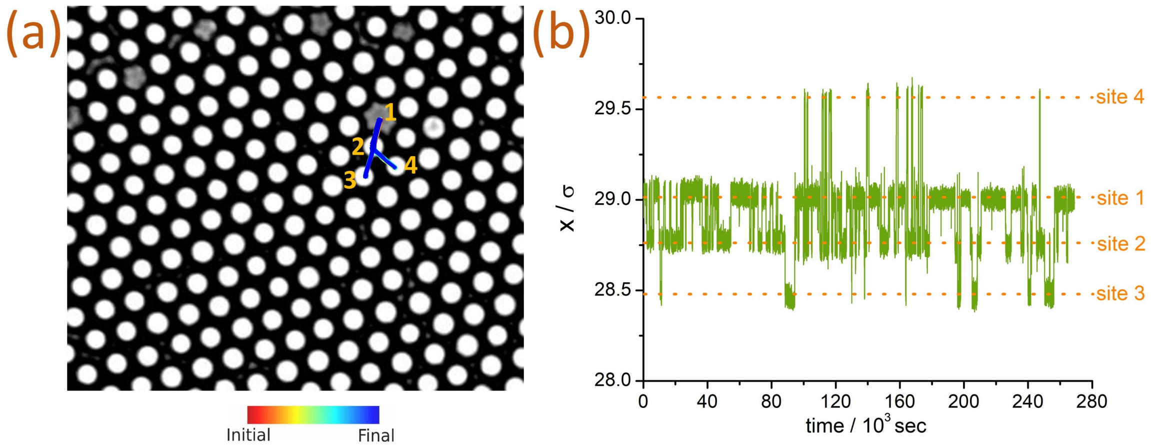

Figure S3: (a) Original experimental photo taken at $t = 0$ for another active but sluggish vacancy, superimposed with trajectory lines drawn with 13443 time segments. This is taken from the same sample of Figure 1 in the main text, which has $\phi = 0.837$ under 3.11 days of observation. (b) shows the movement of the 6-folded symmetric vacancy against time. It has hopped 159 times between 4 lattice sites with $P_{ret} = 79.9\%$. The 6-folded symmetric vacancy is close to the crystalline boundary, which has some 5-folded symmetric point defects. Indeed, 5-folded symmetric point defects are common at the boundaries between our large crystals.

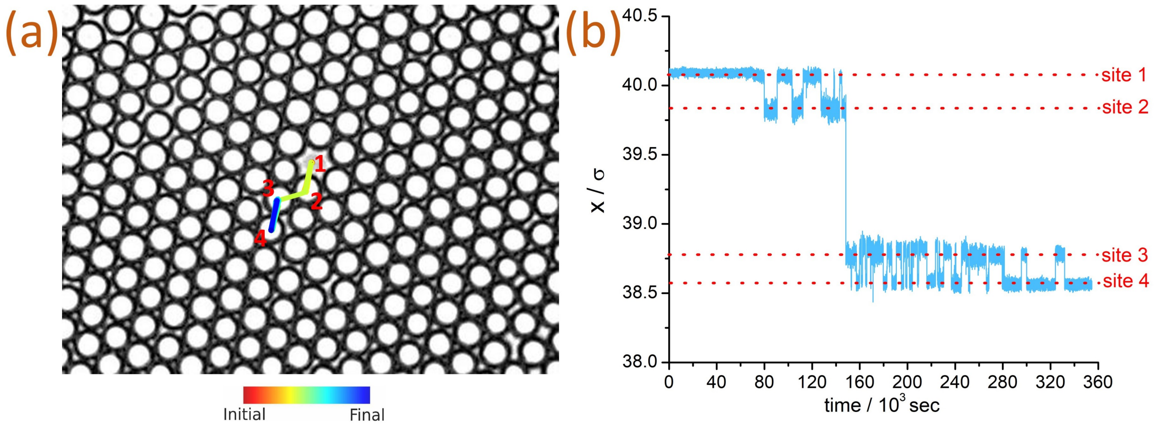

Figure S4: (a) Original experimental photo taken at $t = 0$ for another active but sluggish vacancy, superimposed with trajectory lines drawn with 10370 time segments. This is taken from a sample with $\phi = 0.821$ under 3.84 days of observation. This vacancy is isolated from other point defects or the crystal boundaries. A distortion of the crystal structure is also observed here. (b) shows the vacancy movement against time. The vacancy has hopped 57 times between 4 lattice sites with $P_{ret} = 94.7\%$.

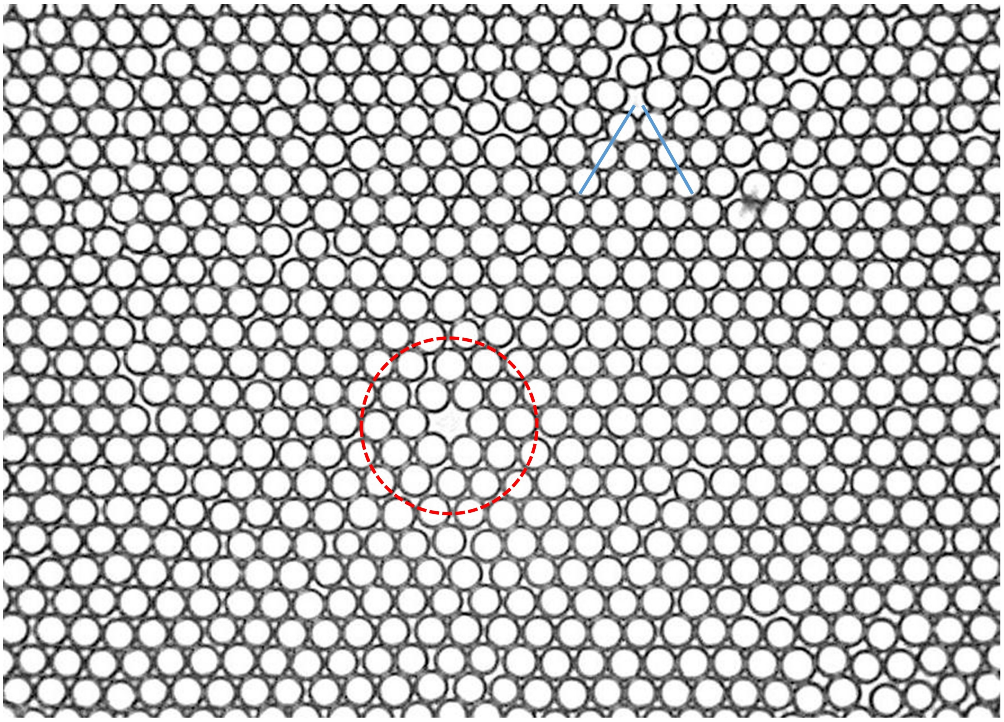

Figure S5: Original experimental photo of another inert vacancy with 6 nearest neighboring particles. It is taken from a sample with $\phi = 0.838$, under a continuous observation time of 4.94 days. It stays at the same site throughout the observation period. The inert vacancy is the same as that in Figure 4 of the main text, but the two photos are taken at different moments. This sample has 3 more similar inert vacancies, which are not further shown. A 5-folded symmetric point defect is also shown in the photo, which is accompanied by two dislocation lines as shown.

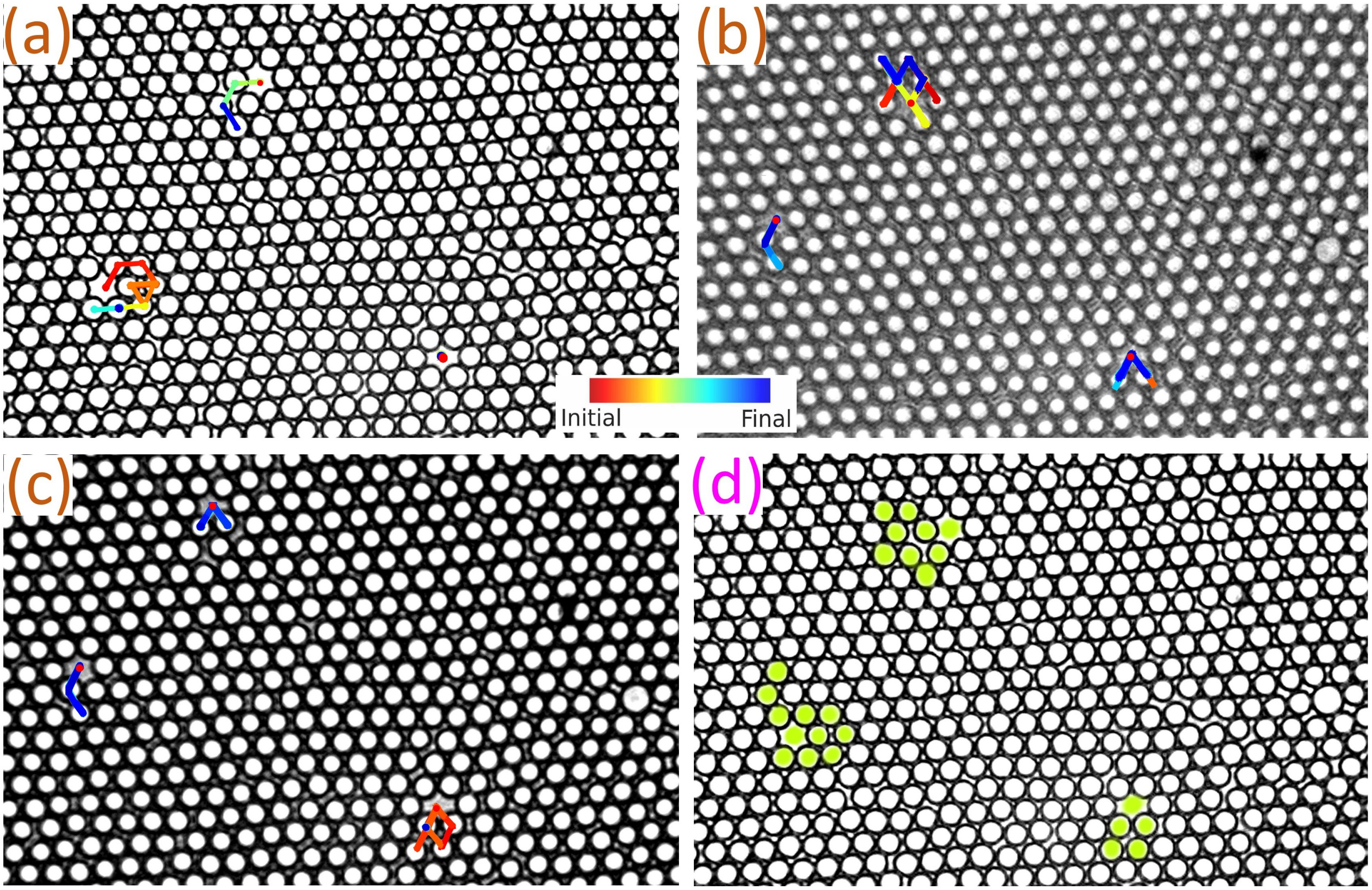

Figure S6: Original experimental photos of the highest density sample with $\phi = 0.840$. The observation is done in three separate periods of 4 days, 4.63 days, and 4.63 days. (a), (b), and (c) are the initial photos of these three periods, superimposed with the vacancy trajectories on them. (d) shows the same experimental photo as in (a), but has highlighted the lattice sites visited by the vacancies throughout the whole observation time. An especially big particle found on the right-hand side of these photos serves as a reference point to compare the vacancy locations in different observation periods. Throughout the 13.26 days of observation, the vacancy in the upper part of this selected region has hopped 341 times with $P_{ret} = 91.2\%$, while the vacancy in the lower right part of this region has hopped 135 times with $P_{ret} = 79.3\%$. The vacancy in the lower left part of this selected region has hopped 900 times with $P_{ret} = 96.6\%$, which is also the highest back-returning hop probability among all our vacancies in different samples.

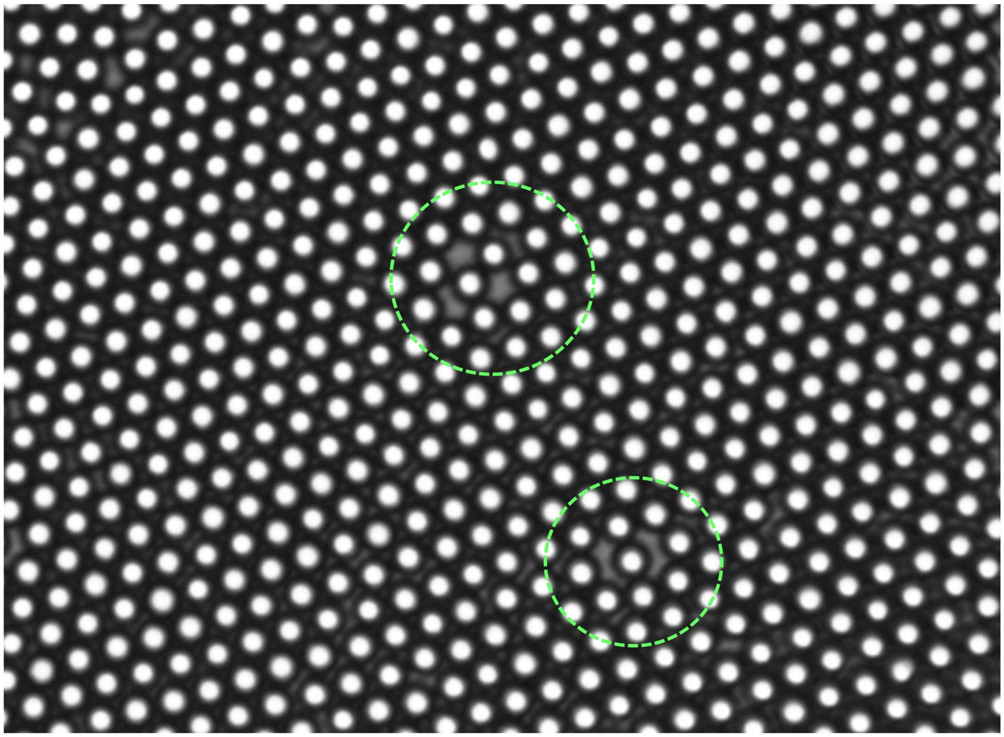

Figure S7: A selected region from the sample with $\phi = 0.801$ demonstrates vacancies in two rare cases. The vacancy circled below is a split SV mono-vacancy. [20] This vacancy form is always seen in the short instanton time when a 6-folded symmetric vacancy is hopping from one lattice site to another neighboring lattice site. Its existence time is usually too short that we do not consider it as a metastable state, which is different from *Pertsinidis and Ling*. [19,20] However, in this rare case, the central particle is trapped by the two nearest neighboring particles below and above it, making this split SV configuration exist for a very long time. The vacancy circled above is a split SD_b di-vacancy in *Pertsinidis and Ling*, [20] or the 2-folded symmetric D_2 dimer vacancy in *Zhang and Liu*. [21] This vacancy is inert throughout our 3.18 days of observation.

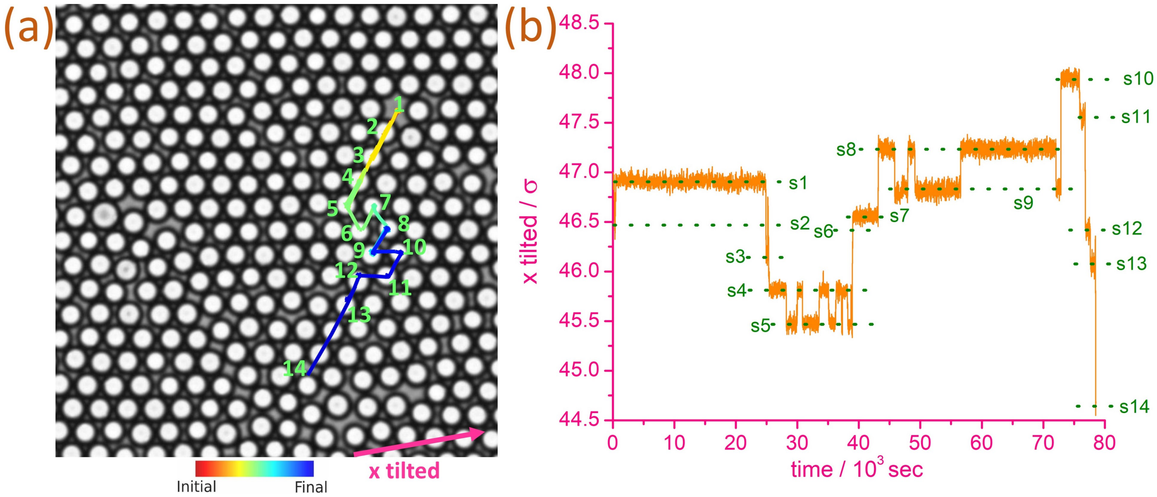

Figure S8: (a) Original experimental photo at $t = 0$ for a vacancy in the sample with $\phi = 0.792$, which is absorbed into the amorphous boundary at around 78532 sec $\lesssim$ 0.91 day. [50,51] Trajectory lines drawn with 3926 time segments are superimposed on the photo to show the moving path. (b) shows its movement against time. It has traveled across 15 lattice sites before reaching the amorphous boundary, making 29 hops with $P_{ret} = 57.1\%$. Sites, where the vacancy has stayed for more than 12 seconds, are marked in (a) and shown as sN in (b).

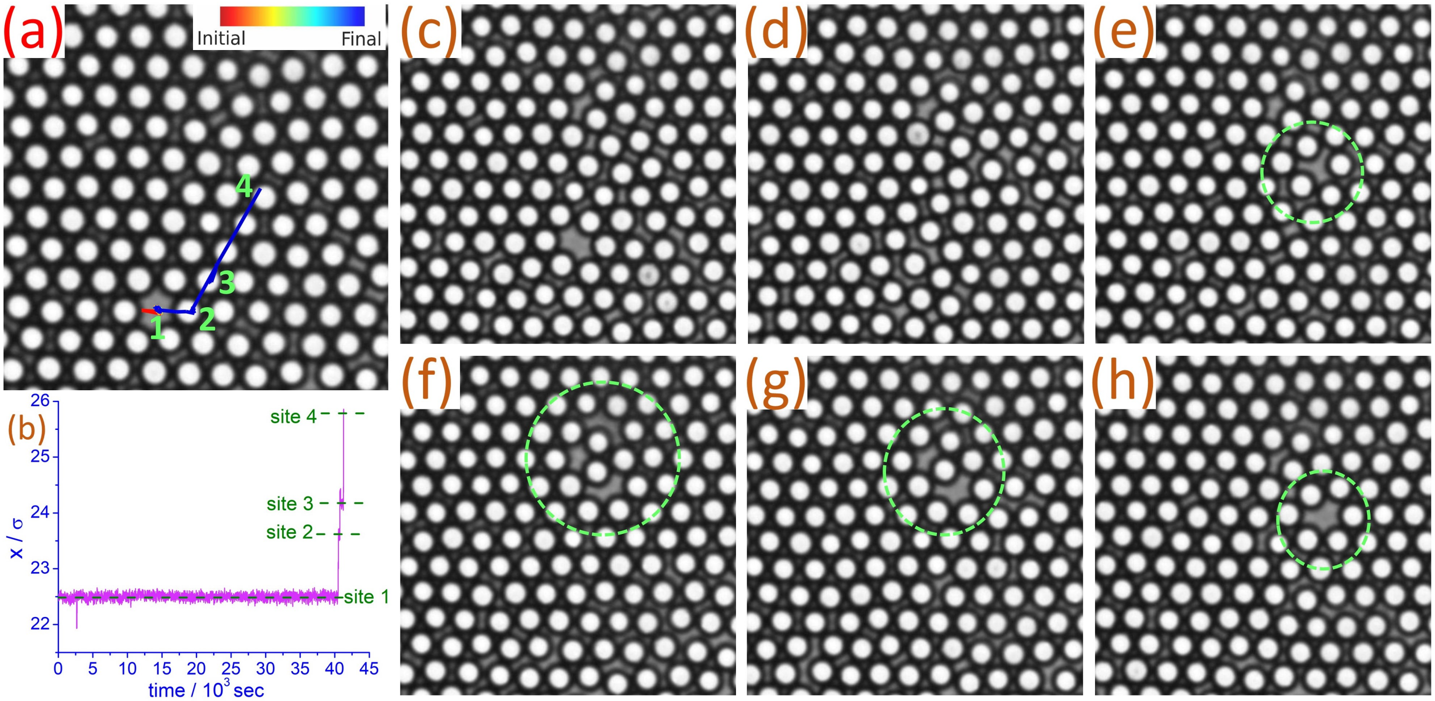

Figure S9: (a) Original experimental photo of a vacancy in the sample with $\phi = 0.792$ at $t = 0$. Trajectory lines with 2065 segments are superimposed on it to show the vacancy movement up to $t = 41300$ sec when the vacancy has finished a big hop from site 3 to site 4. (b) shows the detailed vacancy movement in this period. (c) shows the vacancy in site 3 at $t = 41204$ sec before making the big hop to site 4. (d) shows a moment during the big hop at $t = 41252$ sec. (e) shows the vacancy arrives at site 4 at $t = 41252$ sec. At this time, the vacancy is already not in a form surrounded by 6 nearest neighboring particles. After $t = 41252$ sec, the vacancy mainly exists in three different configurations as represented by 3 selected moments at $t =$ (f) 41340 sec, (g) 41660 sec, and (h) 41912 sec. (f) looks like the di-vacancy in Figure S7, but it has one more particle, which is thus a mono-vacancy. The vacancy later stays in this form for a very long time. (g) is the split SV mono-vacancy which is usually seen in the short instanton time. The vacancy in (h) is surrounded by 6 nearest neighboring particles again.

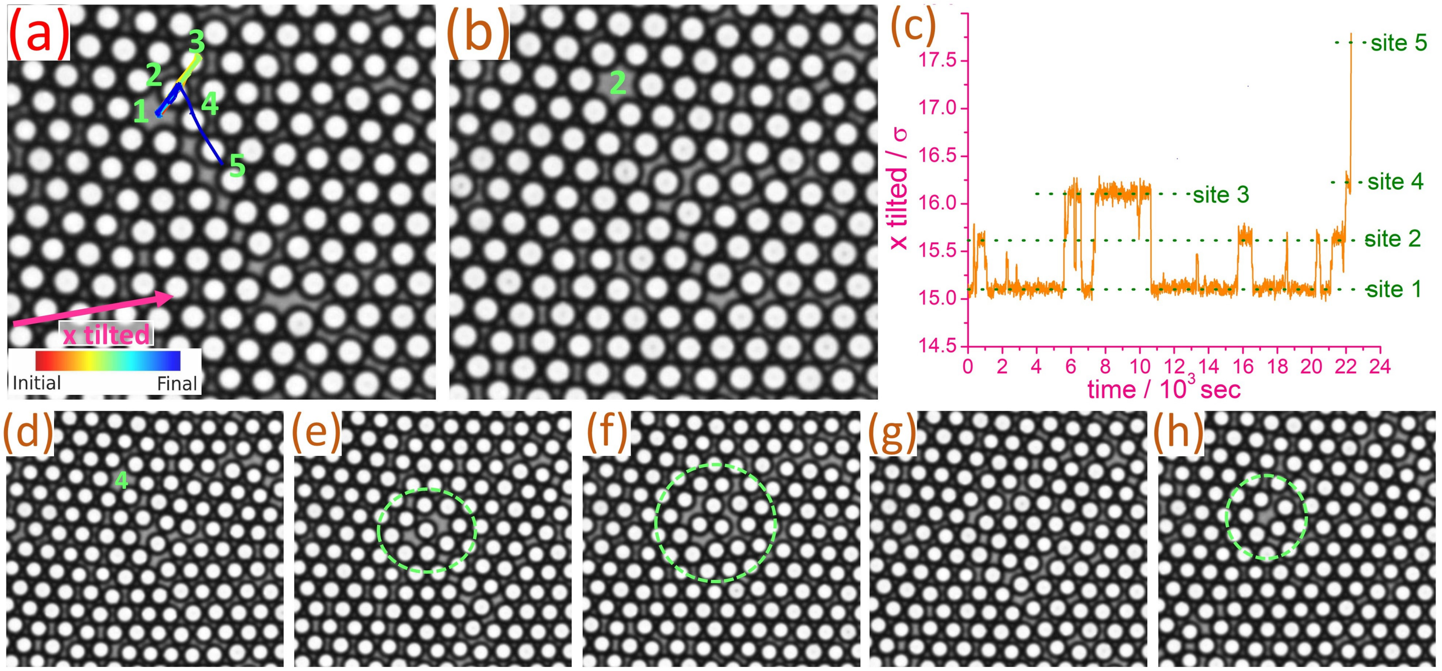

Figure S10: (a) Original experimental photo of a vacancy in the sample with $\phi = 0.792$ at $t = 0$. Trajectory lines with 1115 segments are superimposed on it to show the movement of the vacancy up to $t = 22304$ sec when the vacancy has finished a big hop from site 4 to site 5, and soon gets absorbed. Dislocation is also found in (a), and thus we show in (b) when the crystal is in a better shape at $t = 640$ sec. (c) shows the detailed vacancy motion in this period. (d) shows the vacancy at site 4 before making the big hop at $t = 22240$ sec. (e) shows the vacancy arrives at site 5 and changes to split SV configuration at $t = 22304$ sec. [20] Up to this time, the vacancy has made 23 hops with $P_{ret} = 81.8\%$. It then quickly changes to a similar configuration as found in Figure S9(f) at $t = 22320$ sec. Soon it is absorbed into the background as shown in (g) at $t = 22400$ sec. After that, occasionally vacancy emerges again, and (h) shows one moment at $t = 26320$ sec when the vacancy emerges in a 5-folded symmetric form, and will soon be absorbed again.

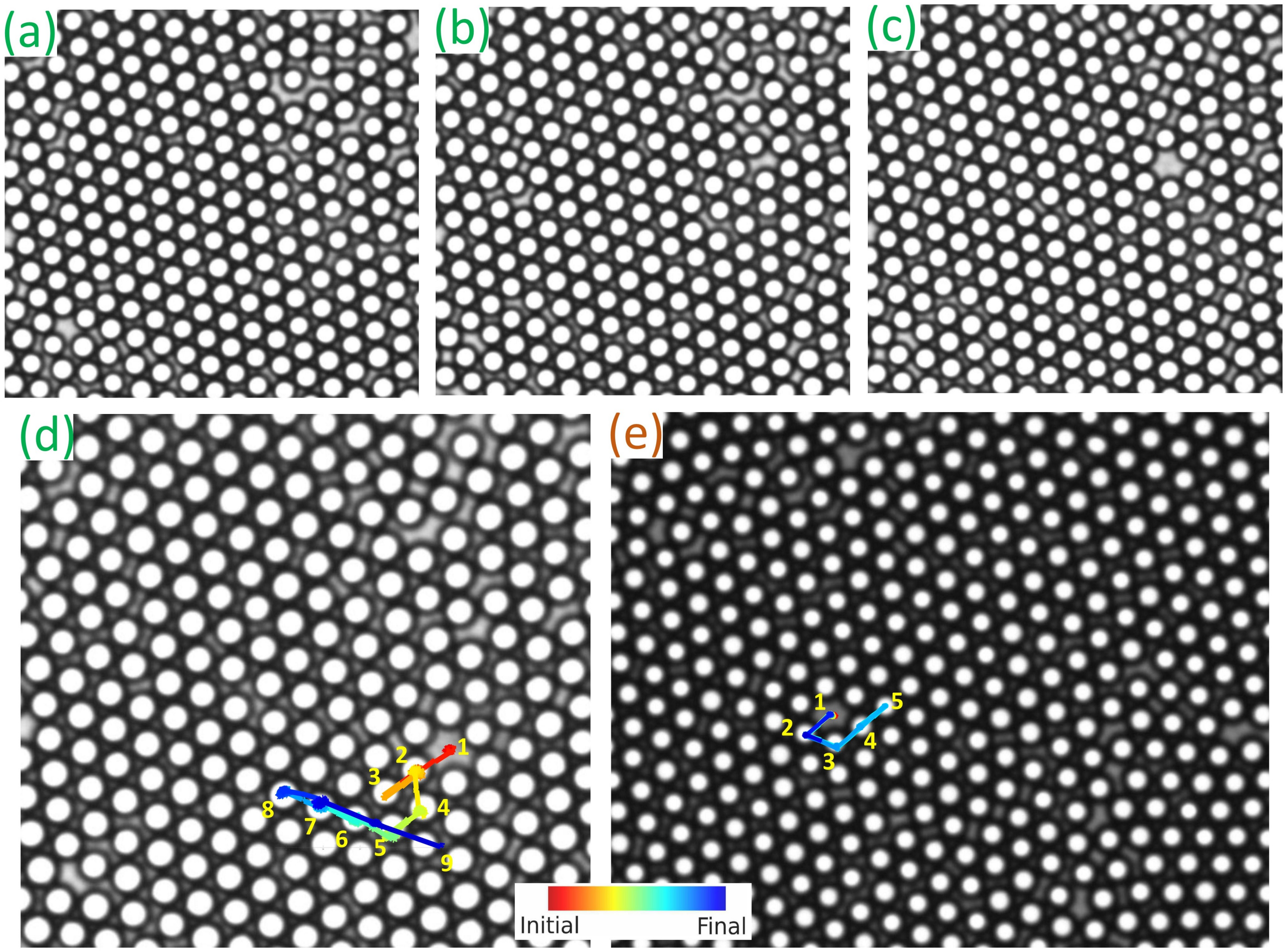

Figure S11: Two selected regions from two different samples in the group of lowest packing fraction (ϕ). Both samples have $\phi = 0.781$. Photos in (a)−(d) show the same selected region of one sample which is observed for around 1.9 days. (a)−(c) demonstrate the formation of a vacancy near the amorphous boundary, with $t =$ (a) 0, (b) 810 sec, and (c) 900 sec. (d) shows the photo at $t = 2060$ sec when the vacancy has already moved downward for a few sites. The subsequent vacancy trajectory for the next 0.21 days, until the vacancy is absorbed into the media again, is drawn on the photo. The vacancy has traveled through 9 sites in this relatively short time and has made 12 hops with $P_{ret} = 33\%$ only. (e) is the initial photo of another sample which is observed for 1.85 days. The vacancy trajectory, which is not taken time coarsening, is superimposed on the photo. The vacancy travels through 5 lattice sites and makes 16 hops with $P_{ret} = 56.3\%$.

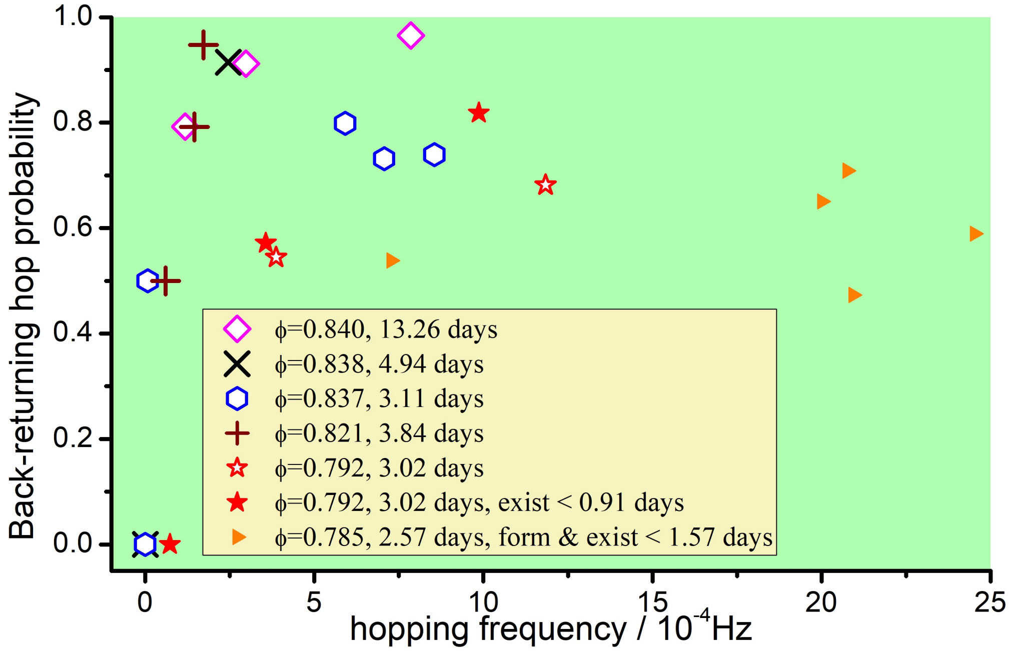

Figure S12: Back-returning hop probabilities (P_{ret}) vs hopping frequencies of selected vacancies in six samples with different packing fractions (ϕ). Vacancies in the same samples hop with different frequencies, i.e. different average waiting time between hopping motions, and different back-return hop probabilities. These demonstrate the heterogeneity in vacancy dynamics. Some vacancies in the samples with low ϕ are formed in the middle of the observation, and are absorbed before the observation ends.

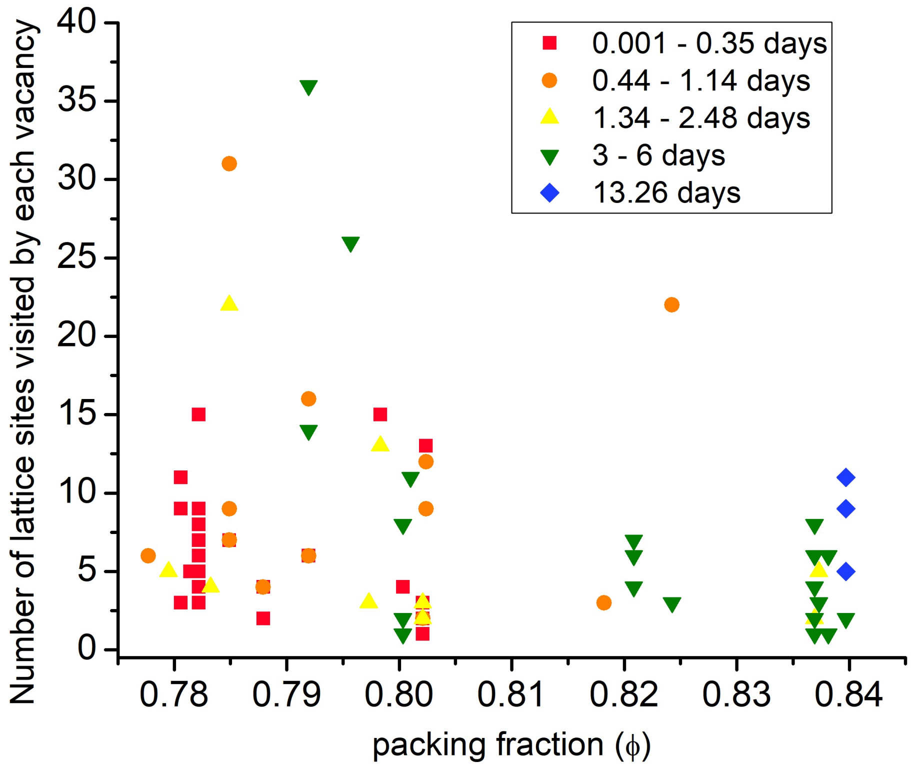

Figure S13: Lattice sites visited by vacancies of the twenty-three samples. The legend shows the observation time of each vacancy. Some vacancies are formed in the middle of the observation, and some disappear before the end of the observation. Therefore, the observation time for different vacancies in the same sample is different. Roughly speaking, vacancies in samples with smaller ϕ can hop more easily across different lattice sites, so that it is easier for them to move across sufficient lattice sites and get absorbed into amorphous boundaries in a short time. Moreover, particles in these samples can also rearrange themselves more easily to absorb the vacancies. These agree with the fact that particles and vacancies in metals and alloys generally have stronger diffusion at higher temperatures. The 13.26 days of observation time for the highest ϕ sample is divided into 3 different periods as stated in Figure S6.

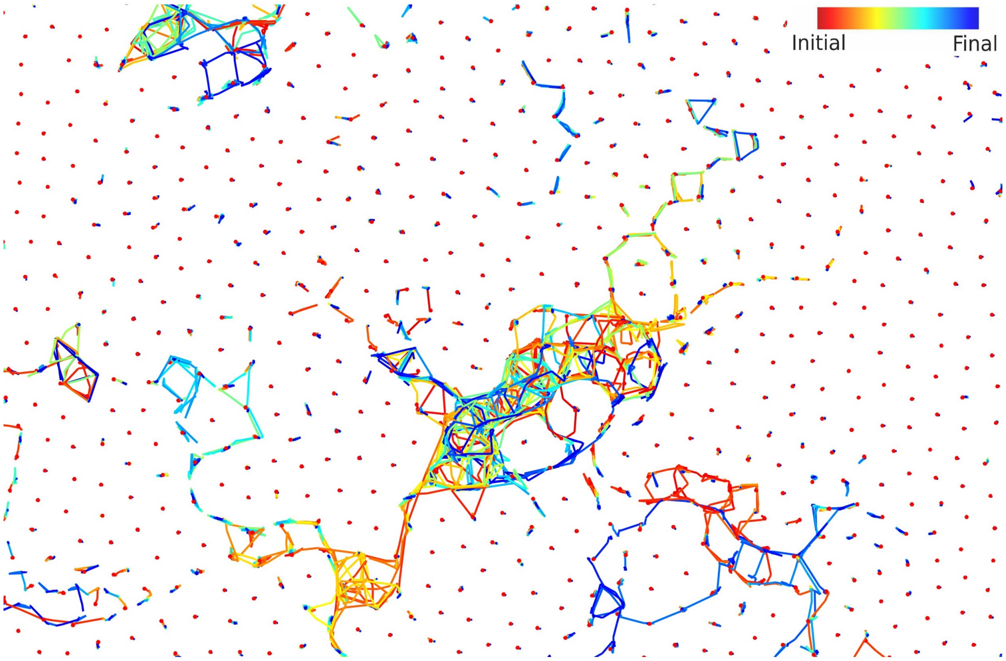

Figure S14: Time coarsening trajectories of a structural disordered colloidal glass demonstrating the dynamical heterogeneity in a glassy system. This is an unpublished figure taken from our previous colloidal glass experiment. [11] This system is composed of particles with a bimodal distribution of particle size. $\phi \approx 0.78$ and the system is observed for 2.69 days. From this figure, some particles are moving strongly around in the localized cooperative rearranging regions (CRR), whereas particles in the other regions can only vibrate around their original locations and are visualized as red dots with little blue patches. This difference in particle dynamics found within the same sample is called dynamical heterogeneity, which is a typical property of glass.

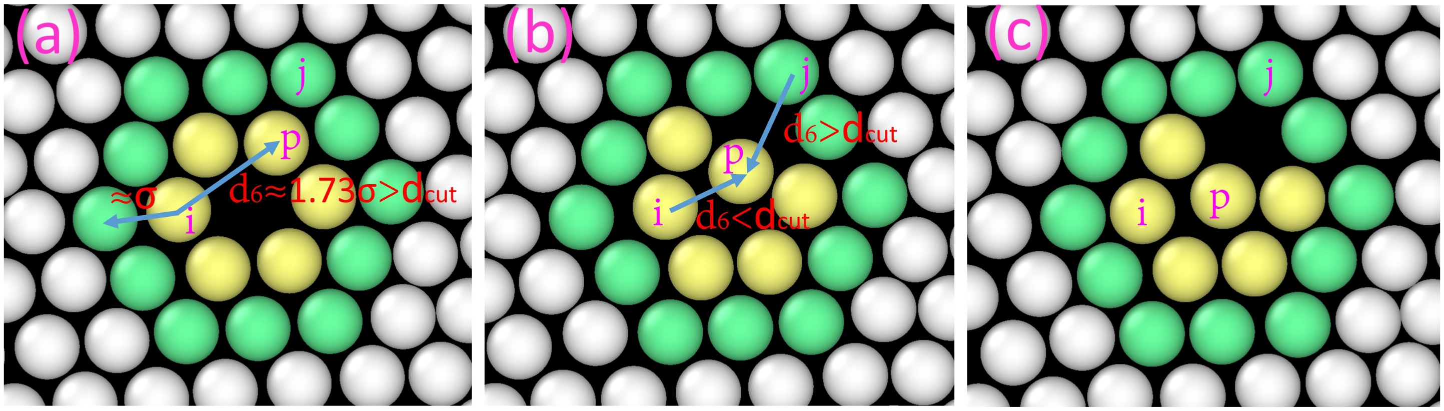

Figure S15: This figure demonstrates the idea of our vacancy tracking code used for the experimental data. One vacancy of our highest density sample ($\phi = 0.840$) is displayed through the visualization software, Ovito. The displayed particle size is tunable in the software. (a), (b), and (c) show the three moments before, at, and after a hopping movement, respectively. The 6 yellow shaded particles are the first neighboring shell of the original vacancy, and their average position is used to define the location of the vacancy before the hop. The green shaded particles are the second neighboring shell of the vacancy. Define d_6 as the separation between a particle and its 6th closest neighbor. Each yellow particle has 5 nearest neighboring particles at around σ apart from it and has $d_6 \approx 1.73\sigma$. By further defining a cutting distance (d_{cut}) to be around 1.3σ, we trace the time when one of the yellow particles has $d_6 < d_{cut}$. Particle i in (a) and (b) illustrate this change. In (b), a particle p is in the middle of an attempt to hop from one lattice site to another, with the vacancy moving in the opposite direction. Meanwhile, some of the green particles, e.g. particle j, are now having $d_6 > d_{cut}$. At a later time as shown in (c), there are only 6 particles with $d_6 > d_{cut}$ again. If each of these 6 particles has 2 other of these 6 particles sitting next to it within d_{cut} for at least 3 successive time frames, then the vacancy is regarded as either having hopped to a new lattice site or having returned to the original lattice site, depending on whether the 6 new particles surrounding the vacancy are the same as the 6 original particles. When the vacancy is moving between two sites as shown in (b), we pick out the most frequently appearing set of particles that have $d_6 > d_{cut}$. In this set of particles, the hopping particle is identified as the one closest to the average position of them. After removing this hopping particle from the set, we usually get 8 particles surrounding the hopping particle during the movement. The average position of these 8 particles is defined as the location of the vacancy in this short instanton time. Minor corrections are further made by comparing the results with the particle motions tracked in Ovito and in the original photos to ensure accuracy. d_{cut} is chosen to a smaller value of 1.28σ for a few vacancies that are more distorted from the 6-folded symmetric form. However, those distorted vacancies also require more correction work. Some vacancies in the medium-density ($\phi \lesssim 0.8$) samples are too distorted that we directly count their back-returning hop statistics by checking their original photos one by one.

References

[11] C.-T. Yip, M. Isobe, C.-H. Chan, S.-M. Ren, K.-P. Wong, Q.-X. Huo, C.-S. Lee, Y.-H. Tsang, Y.-L. Han, C.-H. Lam, *Phys. Rev. Lett.* **2020**, 125, 258001.

[19] A. Pertsinidis, X. S. Ling, *Nature* **2001**, 413, 147.

[20] A. Pertsinidis, X. S. Ling, *Phys. Rev. Lett.* **2001**, 87, 098303.

[21] T. H. Zhang, X. Y. Liu, *Appl. Phys. Lett.* **2006**, 89, 261914.

[28] A. Ninarello, L. Berthier, D. Coslovich, *Phys. Rev. X* **2017**, 7, 021039.

[29] E. R. Weeks, *ACS Macro Lett.* **2017**, 6, 27.

[34] H. Tong, P. Tan, N. Xu, *Sci. Rep.* **2015**, 5, 1.

[42] E. P. George, D. Raabe, R. O. Ritchie, *Nat. Rev. Mater.* **2019**, 4, 515.

[49] The built-in function, imfindcircles, in either Octave or Matlab helps to find the positions and radii of circular objects.

[50] H. Zhang, D. J. Srolovitz, J. F. Douglas, J. A. Warren, *Proc. Natl. Acad. Sci.* **2009**, 106, 7735.

[51] K. H. Nagamanasa, S. Gokhale, R. Ganapathy, A. K. Sood, *Proc. Natl. Acad. Sci.* **2011**, 108, 11323.

[52] A. P. Thompson, H. M. Aktulga, R. Berger, D. S. Bolintineanu, W. M. Brown, P. S. Crozier, P. J. in't Veld, A. Kohlmeyer, S. G. Moore, T. D. Nguyen, R. Shan, M. J. Stevens, J. Tranchida, C. Trott, S. J. Plimpton *Comput. Phys. Commun.* **2021**, 271, 108171.

[53] J. C. Crocker, D. G. Grier, E. Weeks, Particle tracking using IDL, *http://www.physics.emory.edu/faculty/weeks//idl/*, accessed: July 2018.
